# On the clubionid spiders (Araneae, Clubionidae) from Xishuangbanna, China, with descriptions of two new genera and seven new species

**DOI:** 10.3897/zookeys.1062.66845

**Published:** 2021-10-14

**Authors:** Jianshuang Zhang, Hao Yu, Shuqiang Li

**Affiliations:** 1 School of Life Sciences, Guizhou Normal University, Guiyang, Guizhou, China Guizhou Normal University Guiyang China; 2 School of Biological Sciences, Guizhou Education University, Guiyang, Guizhou, China Guizhou Education University Guiyang China; 3 Institute of Zoology, Chinese Academy of Sciences, Beijing, China Institute of Zoology, Chinese Academy of sciences Beijing China

**Keywords:** Aranei, new synonymy, sac spider, taxonomy, tropical rainforest

## Abstract

Clubionid spiders from Xishuangbanna, Yunnan Province, China are studied. A total of seven genera and 13 species have been found, including two new genera with one new species each, i.e., *Ramosatidia* Yu & Li, **gen. nov.**, with *R.situ* Yu & Li, **sp. nov.** (♂♀) as the type species and *Sinostidia* Yu & Li, **gen. nov.**, with *S.shuangjiao* Yu & Li, **sp. nov.** (♂♀) as the type species. Five additional new species are *Sinostidiadujiao* Yu & Li, **sp. nov.** (♂♀), *Matidiaxieqian* Yu & Li, **sp. nov.** (♂♀), *Nusatidiachangao* Yu & Li, **sp. nov.** (♂♀), *N.mianju* Yu & Li, **sp. nov.** (♀), and *N.subjavana* Yu & Li, **sp. nov.** (♀). The following genera and species are reported from China for the first time: *Malamatidia* Deeleman-Reinhold, 2001, *Pteroneta* Deeleman-Reinhold, 2001, *Malamatidiazu* Jäger & Dankittipakul, 2010, *Nusatidiaaeria* (Simon, 1897), *N.camouflata* Deeleman-Reinhold, 2001, *Porrhoclubionapteronetoides* (Deeleman-Reinhold, 2001), and *Pteronetaultramarina* (Ono, 1989). *Malamatidiachristae* Jäger & Dankittipakul, 2010 **syn. nov.** is a junior synonym of *Malamatidiazu. Nusatidiarama* Deeleman-Reinhold, 2001 **syn. nov.** is synonymised with *N.aeria* (Simon, 1897).

## Introduction

The Clubionidae Wagner, 1887 is a relatively large family with 656 valid species distributed worldwide (WSC 2021), and 517 of these are placed in the genus *Clubiona* Latreille, 1804 (WSC 2021). Before the current study, 178 species in five genera (Li 2020) were known from China, with 172 species assigned to *Clubiona*. Of these, 51 *Clubiona* species have been recorded from Xishuangbanna, with 30 described as new species in the last three years (Yu and Li 2019a, b; Zhang et al. 2021). Herein, we discuss 13 species in seven genera of Clubionidae found in Xishuangbanna, including two genera with seven species new to science, and two genera with five species new to China.

## Materials and methods

Specimens were primarily collected by canopy fogging, while a few were obtained by beating vegetation and pitfall trapping. All type specimens are deposited in the Institute of Zoology, Chinese Academy of Sciences (**IZCAS**, curator Jun Chen) in Beijing, China.

Specimens were examined using a LEICA M205C and an Olympus SZX7 stereomicroscope. Further details were studied under a CX41 compound microscope. Male and female genitalia were examined and illustrated after dissection. Left male palps are illustrated, unless otherwise indicated, and photos of the right palps are flipped horizontally in figures to allow ease of comparison with other species. Epigynes were removed and cleared in lactic acid or warm 10% potassium hydroxide (KOH) solution before illustration. Vulvae were imaged after being embedded in Arabic gum (Yuanye Biotechnology Co., Ltd). Images were captured with a Canon EOS 70D digital camera (20.2 megapixels) mounted on an Olympus CX41 compound microscope and assembled using Helicon Focus 6.80 image stacking software. All measurements were obtained using an Olympus SZX7 stereomicroscope and are given in millimetres. Eye diameters are measured at the widest points. The total body length does not include the chelicerae or spinnerets. Leg lengths are given as total length (femur, patella+tibia, metatarsus, tarsus).

References to figures in the cited papers are listed in lowercase (fig. or figs); figures from this paper are noted with an initial capital (Fig. or Figs). Abbreviations used in the text and figures are as follows:

**A** epigynal atrium;

**AER** anterior eye row;

**ALE** anterior lateral eyes;

**AME** anterior median eyes;

**BS** bursa;

**C** conductor;

**CD** copulatory duct;

**CO** copulatory opening;

**DTA** dorsal tibial apophysis;

**E** embolus;

**EB** embolic base;

**EPP** epigynal posterior plate;

**FD** fertilisation duct;

**LTA** lateral tibial apophysis;

**MOQ** median ocular quadrangle;

**MOQL** length of MOQ;

**MOQA** anterior width of MOQ;

**MOQP** posterior width of MOQ;

**PER** posterior eye row;

**PLE** posterior lateral eyes;

**PME** posterior median eyes;

**PTA** prolateral tibial apophysis;

**RTA** retrolateral tibial apophysis;

**SB** spermathecal base;

**SH** spermathecal head;

**SP** spermatheca;

**TA** tegular apophysis;

**TG** tegular groove;

**TU** tutaculum;

**VTA** ventral tibial apophysis.

Most of the terminology used in the text and figure legends follows Yu and Li (2019a, b), and some follows Deeleman-Reinhold (2001) and Zhang et al. (2018).

DNA barcodes were obtained for species delimitation and matching of sexes. A partial fragment of the mitochondrial cytochrome oxidase subunit I (CO1) gene was amplified and sequenced for 21 specimens using the primers LCOI1490 (5'-GGTCAACAAATCATAAAGATATTG-3') and HCOI2198 (5'-TAAACTTCAGGGTGACCAAAAAAT-3'). For additional information on extraction, amplification, and sequencing procedures, see Malumbres-Olarte and Vink (2012).

Sequences were trimmed to 656 bp. All sequences were confirmed using BLAST and are deposited in GenBank. The codes and GenBank accession numbers of voucher specimen are provided as follows: *Malamatidiazu*: YHCLU0119, ♂, GenBank MZ508477; YHCLU0120, ♀, GenBank MZ508476. *Matidiaspatulata* Chen & Huang, 2006: YHCLU0045, ♂, GenBank MZ508480; YHCLU0046, ♀, GenBank MZ508479. *M.xieqian* sp. nov.: YHCLU0126, ♂, GenBank MZ508472; YHCLU0127, ♀, GenBank MZ508471. *Nusatidiaaeria*: YHCLU0150, ♂, GenBank MZ508463; YHCLU0149, ♀, GenBank MZ508464. *N.changao* sp. nov.: YHCLU0152, ♂, GenBank MZ508461; YHCLU0129, ♀, GenBank MZ508470. *N.mianju* sp. nov.: YHCLU0131, ♀, GenBank MZ508469. *N.subjavana* sp. nov.: YHCLU0123, ♀, GenBank MZ508475. *Porrhoclubionapteronetoides*: YHCLU0124, ♂, GenBank MZ508474; YHCLU0125, ♀, GenBank MZ508473. *Pteronetaultramarina*: YHCLU0136, ♂, GenBank MZ508466; YHCLU0137, ♀, GenBank MZ508465. *Ramosatidiasitu* sp. nov.: YHCLU0134, ♀, GenBank MZ508467. *Sinostidiadujiao* sp. nov.: YHCLU0132, ♂, GenBank MZ508468; YHCLU0090, ♀, GenBank MZ508478. *S.shuangjiao* sp. nov.: YHCLU0151, ♂, GenBank MZ508462; YHCLU0155, ♀, GenBank MZ508460. We were unable to obtain good extractions from *Nusatidiacamouflata* Deeleman-Reinhold, 2001, and the male of *Ramosatidiasitu* sp. nov.

## Taxonomy

**Family Clubionidae Wagner, 1887**

### Key to clubionid genera occurring in East and Southeast Asia

(The key refers to specimen colour in ethanol, unless indicated)

**Table:** 

1	Carapace flat and wide, slightly narrowed towards the head (Deeleman-Reinhold 2001: figs 100, 104); small (3–4 mm) (Fig. 18E–H)	**2**
–	Carapace not so flat, narrowed towards the head; medium to large, usually larger than 3.5 mm (Figs 2E–H, 4E–H, 6E–H, 8E–H, 10A–C, 12E–H, 13F, G, 14F, G, 16E–H, 20E–H, 22E–H, 24E–H)	**4**
2	Tarsi II without scopula (also called a ‘flag’ or ‘brush’ by some authors, represented by a dense row of flattened setae); thoracic groove absent (Deeleman-Reinhold 2001: figs 100, 104)	***Simalio***
–	Tarsi II with peculiar scopula (Deeleman-Reinhold 2001: fig. 115); thoracic groove present (Fig. 18E, G)	**3**
3	Pale yellow or greenish, ventrally without distinct pattern; anterior legs with lyriform scopula on tarsi and metatarsi (Deeleman-Reinhold 2001: fig. 128); male chelicerae unmodified, PME and PLE close together (Deeleman-Reinhold 2001: fig. 123); sperm duct forming two loops in the tegulum (Deeleman-Reinhold 2001: fig. 124)	***Scopalio***
–	Pale green, patterned body marked with lazulite blue spots (Fig. 18E–H); feathery scopula occupies only tarsi II (Deeleman-Reinhold 2001: fig. 128); male chelicerae well developed and protruding, dorsally with several spines (Fig. 18E, F), PME and PLE separated by > their diameter (Fig. 18E, G); sperm duct simple, U-shaped in ventral view (Fig. 17D)	***Pteroneta***
4	Living spider yellow or brownish; cephalic part wide; legs short, femur I no longer than carapace width; abdomen oval (Fig. 16E–H)	**5**
–	Living spider greenish; frail in appearance, with a slender body; cephalic part < 2/3 of carapace width; legs long, femur I usually longer than carapace width; abdomen cylindrical, lanceolate, or V-shaped (Figs 2E–H, 4E–H, 6E–H, 8E–H, 10A–C, 12E–H, 13F, G, 14F, G, 20E–H, 22E–H, 24E–H)	**6**
5	Cymbium with tutaculum, tegular groove serving as a kind of conductor for the embolus, subtegulum small and posteriorly located (Fig. 15A–E); epigyne with large, close or partly fused copulatory openings and 2 thick, hyaline copulatory ducts (Fig. 16A–D)	***Porrhoclubiona***
–	Copulatory organs variable but not as above	***Clubiona* sensu lato**
6	Head width ~ 1/2 of carapace width, leg formula 1423, venter of abdomen with dark spot in males (Figs 4E–H, 6E–H)	***Matidia***
–	Head wider than half of carapace width, leg I is not longest, male abdomen ventrally without dark spot (Figs 2E–H, 8E–H, 10A–C, 12E–H, 13F, G, 14F, G, 20E–H, 22E–H, 24E–H)	**7**
7	Body bottle-green (Fig. 20E–H), male with only one promarginal tooth, female without retromarginal tooth; male palpal tibia with four apophyses (Fig. 19A, B); copulatory ducts covered by bursae in dorsal view (Fig. 20A–D)	***Ramosatidia* gen. nov. (*R.situ* sp. nov.)**
–	Body pale yellow, white or brownish (Figs 2E–H, 8E–H, 10A–C, 12E–H, 13F, G, 14F, G, 22E–H, 24E–H); both promargin and retromargin with at least two teeth; male palpal tibia with one or two apophyses (Figs 1B, 7B, 9B, 11B, 21B, 23B); in dorsal view, copulatory ducts are distinct and not covered by spermathecae or bursae (Figs 2C, D, 8C, D, 12C, D, 13D, E, 14D, E, 22C, D, 24C, D)	**8**
8	Cheliceral promarginal teeth closer to the fang than retromarginal ones; male palpal tibia with 2 apophyses (Figs 21B, 23B); epigynal plate with large, shallow depression (or atrium) as broad as epigyne, spermathecae with subglobular head and twisted base (Figs 22A–D, 24A–D)	***Sinostidia* gen. nov.**
–	Cheliceral promarginal teeth further from the fang than the retromarginal teeth, or equidistant (Yu et al. 2017: fig. 9); male palpal tibia with retrolateral apophysis only (Figs 1B, 7B, 9B, 11B); epigyne not as above (Figs 2A–D, 8A–D, 12A–D, 13A–E, 14A–E)	**9**
9	Free part of embolus claw-like, comparatively short, shorter than 1.5× embolic base (Deeleman-Reinhold 2001: figs 185, 190, 194, 200; Zhang et al. 2020: figs 3B, D–F, 4A–C); epigynal plate a large disc, posterior margin heavily sclerotised, spermathecae and bursae (usually pigmented and sclerotised) distinctly visible through integument (Deeleman-Reinhold 2001: figs 187, 188, 191, 192, 195, 196, 202; Zhang et al. 2020: fig. 2A–F)	***Pristidia***
–	Free part of embolus filamentous or thread-like, relatively long, longer than 1.5× embolic base, distally draped around the tegulum (Figs 1C–E, 7C–E, 9C–E, 11C–E); epigynal plate not as above, posterior margin not always sclerotised, bursae (usually hyaline and membranous, sometimes reduced) often obscured through epigynal plate in ventral view (Figs 2A, 8A, 12A, 13A, 14A)	**10**
10	Sternum without rectangular extension beyond coxae I; filiform embolus curving along the tegular distal margin (Fig. 1C, D), sperm duct forming an S-shaped loop and abruptly narrowed (unclear in *M.thorelli*) (Fig. 1D, E); epigyne with a central depression and a median septum, copulatory openings located centrally, covered by hood (Fig. 2A, B)	***Malamatidia***
–	Sternum with rectangular extension anterior to coxae I (Fig. 10B; Deeleman-Reinhold 2001: fig. 152); sperm duct course variable, not abruptly narrowed; central depression and median septum absent, copulatory openings located posteriorly or laterally (Figs 8A, B, 12A, B, 13A, B, 14A, B)	**11 (*Nusatidia*)**
11	Embolus shorter than half of tegulum, its free part slightly curved, tip directed antero-retrolaterally; tegular apophysis present; sperm duct bulky and twisted; RTA concave with two branches (Fig. 7A–E); epigyne ventrally with a circular or square posterior plate; copulatory ducts short and not convoluted; spermathecae large and elongate, bursae reduced or absent (Fig. 8A–D); metatarsi I and II with a pair of dorso-basal spines	***N.aeria* and *N.luzonica***
–	Embolus equal to or longer than tegulum length, typically oriented clockwise along the margin of the tegulum, or draped around the tegulum; tegular apophysis reduced or absent, or represented by a membranous prolongation; RTA simple, unbranched; epigyne without posterior plate; copulatory ducts relatively long, usually convoluted; both spermathecae and bursae present, both subglobular (Figs 12A–D, 13A–E, 14A–E); metatarsi I and II without dorsal spines	***Nusatidia* (excluding *N.aeria* and *N.luzonica*)**

#### Genus Malamatidia

Taxon classificationAnimaliaAraneaeClubionidae

Deeleman-Reinhold, 2001

4468E4EE-6FB2-5FEC-AC78-F27B898D49A9

Malamatidia Deeleman-Reinhold, 2001: 191.

##### Type species.

*Malamatidiabohorokensis* Deeleman-Reinhold, 2001 from Sumatra, Borneo.

##### Diagnosis.

**Figure 1.**
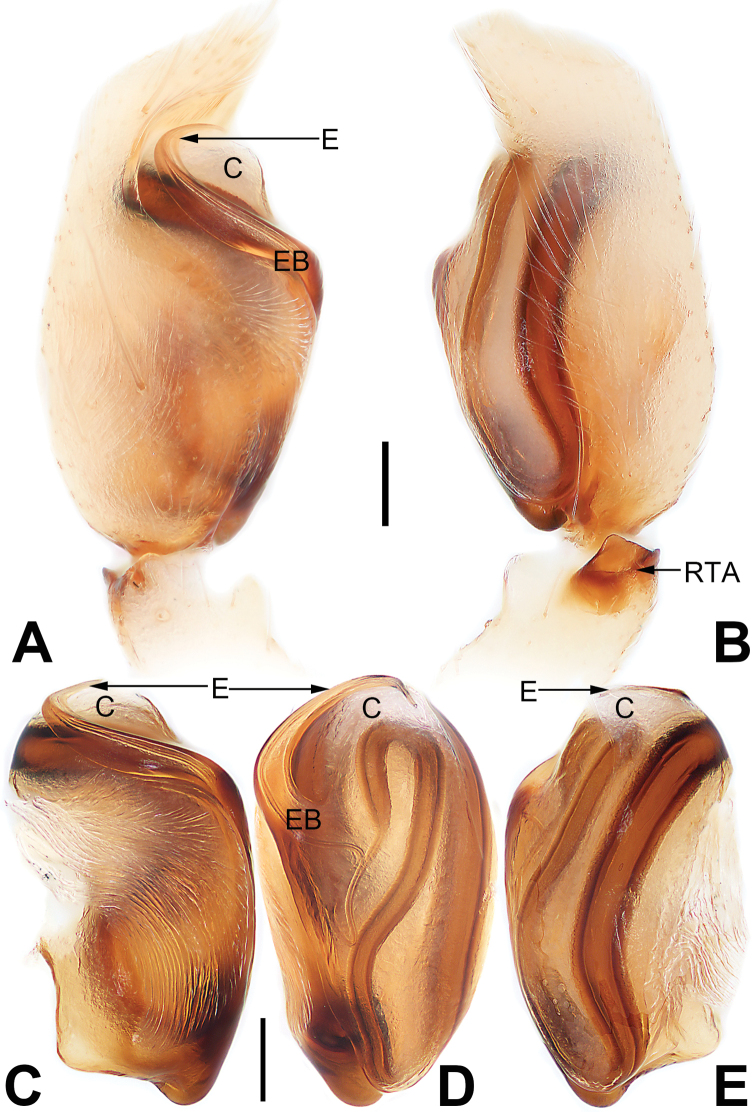
Male palp of *Malamatidiazu***A** prolateral view **B** retrolateral view **C** bulb, prolateral view **D** bulb, ventral view **E** bulb, retrolateral view. Abbreviations: C = conductor; E = embolus; EB = embolic base; RTA = retrolateral tibial apophysis. Scale bars: 0.10 mm (equal for **A, B**, equal for **C–E**).

**Figure 2.**
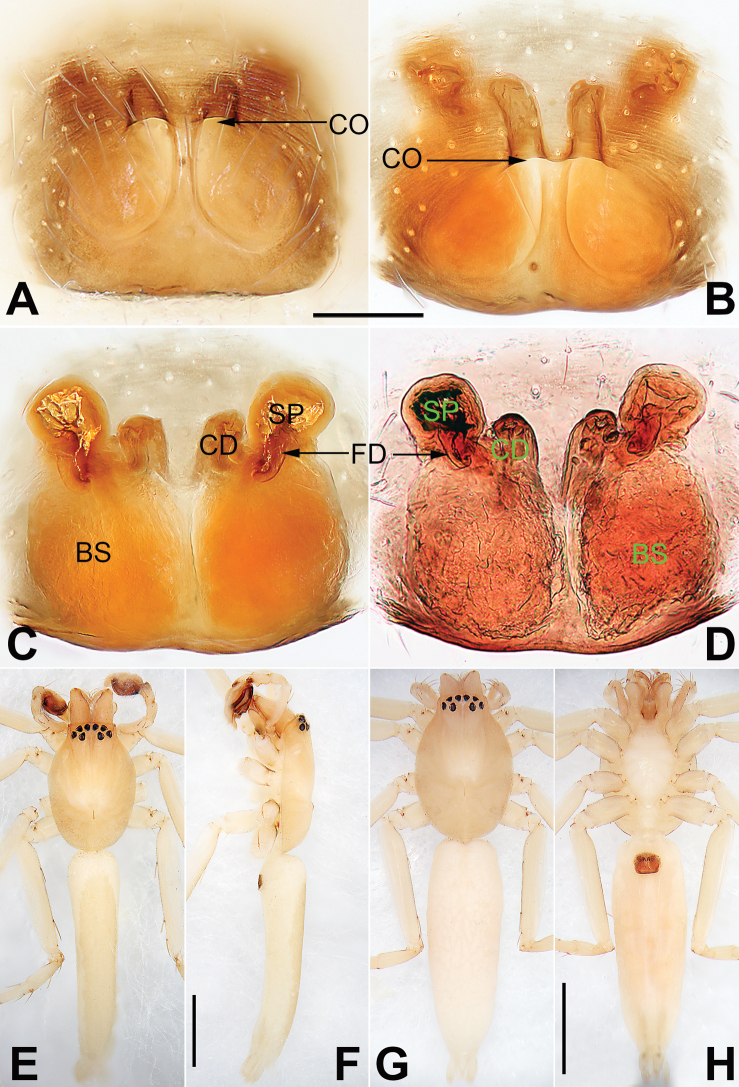
*Malamatidiazu*, epigyne (**A–D**), male habitus (**E, F**) and female habitus (**G, H**) **A** intact, ventral view **B** cleared, ventral view **C** cleared, dorsal view **D** cleared, dorsal view **E** dorsal view **F** lateral view **G** dorsal view **H** ventral view. Abbreviations: BS = bursa; CD = copulatory duct; CO = copulatory opening; FD = fertilization duct; SP = spermatheca. Scale bars: 0.10 mm (equal for **A–D**); 1 mm (equal for **E, F**, equal for **G, H**).

*Malamatidia* resembles *Matidia* Thorell, 1878 and *Nusatidia* Deeleman-Reinhold, 2001 by the long, slender, pale yellow or greenish (in alcohol) body (Figs 2E–H, 4E–H, 6E–H, 8E–H, 10A–C), but it can be distinguished from *Matidia* by the higher cephalic region/carapace width ratio (~ 2/3 vs. 1/2 in *Matidia*) (cf. Figs 2E, G and 4E, G, 6E, G) and femur I is shorter than femur II. It differs from *Nusatidia* by the sternum without a rectangular extension (cf. Figs 2H and 10B). *Malamatidia* species also can be recognised by the copulatory organs: RTA with a sub-rectangular tip (Fig. 1B), filiform embolus curving clockwise and following along the tegular distal margin (Fig. 1C, D), sperm duct an S-shaped loop and sometimes abruptly narrowed (Fig. 1D, E); epigynal plate has a central depression with a longitudinal rim or septum centrally (Fig. 2A, B). All characters of *Malamatidia*, *Nusatidia*, and *Matidia* are according to Deeleman-Reinhold (2001) and Jäger and Dankittipakul (2010).

##### Description.

See Deeleman-Reinhold (2001).

##### Comments.

Based on morphological characters, the genus is probably closely related to *Matidia* and *Nusatidia*. However, the monophyly and the exact placement of *Malamatidia* within Clubionidae remains to be tested.

#### Malamatidia zu

Taxon classificationAnimaliaAraneaeClubionidae

Jäger & Dankittipakul, 2010

D2CD3FA1-93C1-5117-952C-8E59D4394A4A

Figs 1 , 2

Malamatidia zu Jäger & Dankittipakul, 2010: 38, figs 61–63, 73 (♂).
Malamatidia christae Jäger & Dankittipakul, 2010: 39, figs 64–70, 74 (♀). syn. nov.

##### Material examined.

China: **Yunnan**: Xishuangbanna: Mengla County: Xishuangbanna Nature Reserve: 1♂ (YHCLU0119), Xiaolongha biodiversity preservation corridor (21°24.798'N, 101°37.880'E, 690 m), 28 June 2012, G. Zheng leg., Menglun Town: Menglun Nature Reserve: 1♀ (YHCLU0120), primary tropical seasonal rain forest (21°57.883'N, 101°12.147'E, 930 m), 15 August 2011, Q. Zhao leg.; 2♂, primary tropical seasonal rainforest (21°57.669'N, 101°11.893'E, 790 m), 7 August 2007, G. Zheng leg.; 6♀, valley tropical seasonal rainforest (21°54.894'N, 101°16.554'E, 569 m), 1 December 2009, G. Tang leg.

##### Diagnosis.

Males can be characterised by the sickle-shaped embolus, its tip terminated antero-mesally (Fig. 1C, D) (vs. embolus filiform or flagelliform, terminated postero-retrolaterally in other *Malamatidia* spp.). The female of *M.zu* is easily differentiated from all other congeners by the oval bursae (Fig. 2C, D) (vs. globular bursae).

##### Description.

See Jäger and Dankittipakul (2010). Male palp as in Fig. 1A–E, epigyne as in Fig. 2A–D, habitus as in Fig. 2E–H.

##### Comments.

Both sexes were known for all *Malamatidia* species except *M.christae* and *M.zu*. These two species were described based on holotypes from Laos. The former was collected from Luang Nam Tha Province, while the latter was from Luang Prabang Province. Jäger and Dankittipakul (2010) described these two specimens as separate species because of their different habitats, different sizes, and different colours. Recently, new material containing both sexes has been collected from Xishuangbanna. We matched the females and males based on morphological characters (Fig. 2E–H) and DNA barcoding. Consequently, *M.christae* is synonymised with *M.zu*.

##### Distribution.

Laos, China (Yunnan Province, new record). The new collections extend the known range of this species by ~ 250 km to the northwest (Xishuangbanna) from the type locality (Luang Prabang).

#### Genus Matidia

Taxon classificationAnimaliaAraneaeClubionidae

Thorell, 1878

C80FAB48-1D57-52D1-82F3-5B3C889D61A7

Matidia Thorell, 1878: 182.
Kakaibanoides Barrion & Litsinger, 1995: 149 (type K.paranga Barrion & Litsinger, 1995, considered as junior synonym of Matidia by Deeleman-Reinhold, 2001: 156).

##### Type species.

*Matidiavirens* Thorell, 1878 from Moluccas, Sulawesi.

##### Diagnosis.

**Figure 3.**
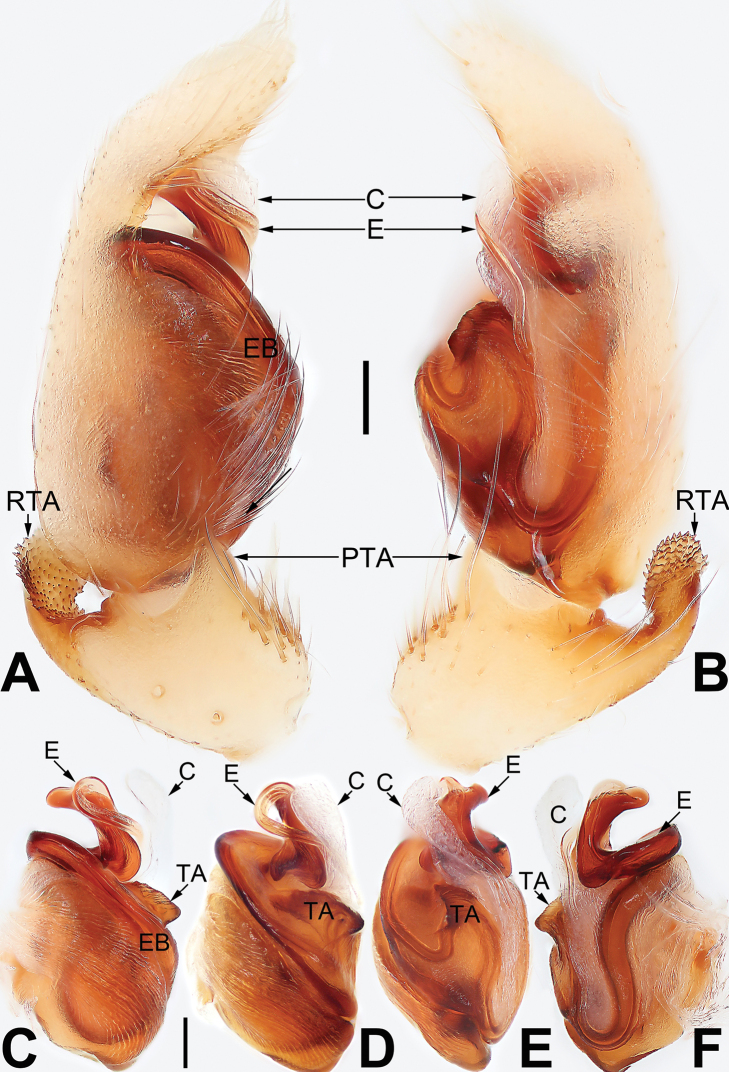
Male palp of *Matidiaspatulata***A** prolateral view **B** retrolateral view **C** bulb, prolateral view **D** bulb, ventral view **E** bulb, ventrolateral view **F** bulb, retrolateral view. Abbreviations: C = conductor; E = embolus; EB = embolic base; PTA = prolateral tibial apophysis; RTA = retrolateral tibial apophysis; TA = tegular apophysis. Scale bars: 0.10 mm (equal for **A, B**, equal for **C–F**).

**Figure 4.**
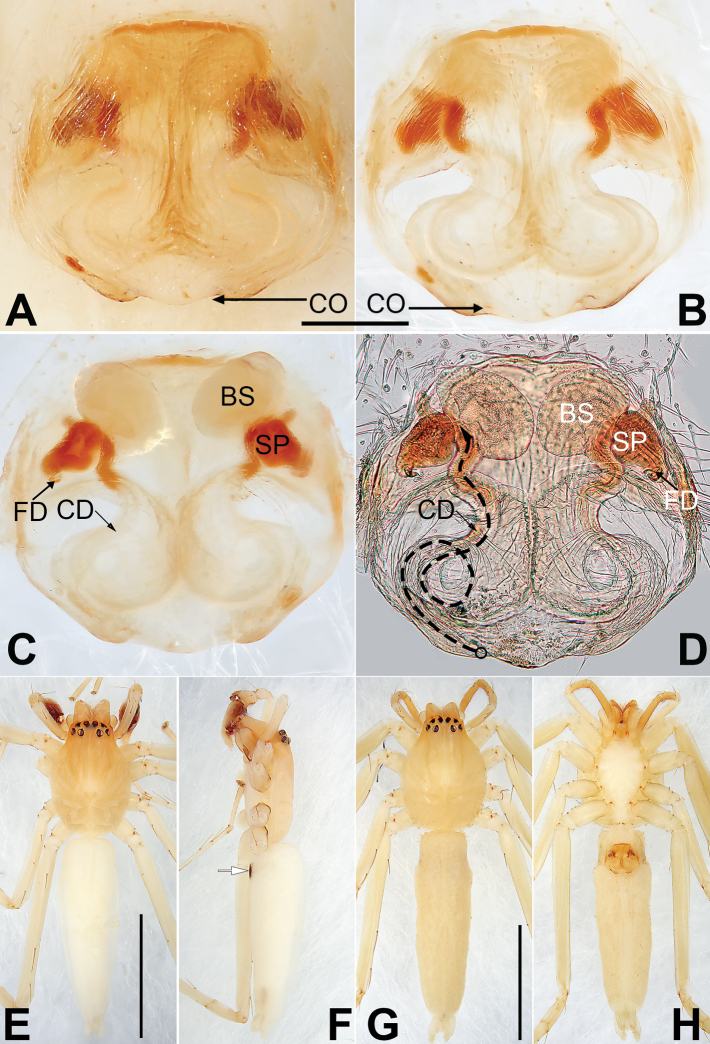
*Matidiaspatulata*, epigyne (**A–D**), male habitus (**E, F**) and female habitus (**G, H**) **A** intact, ventral view **B** cleared, ventral view **C** cleared, dorsal view **D** cleared, dorsal view; path of copulatory duct marked **E** dorsal view **F** lateral view **G** dorsal view **H** ventral view. Arrow (**F**) point at dark ventral abdominal spot in male. Abbreviations: BS = bursa; CD = copulatory duct (dashed line showing schematic course of copulatory duct, dorsal); CO = copulatory opening; FD = fertilization duct; SP = spermatheca. Scale bars: 0.10 mm (equal for **A–D**); 2 mm (equal for **E, F**, equal for **G, H**).

**Figure 5.**
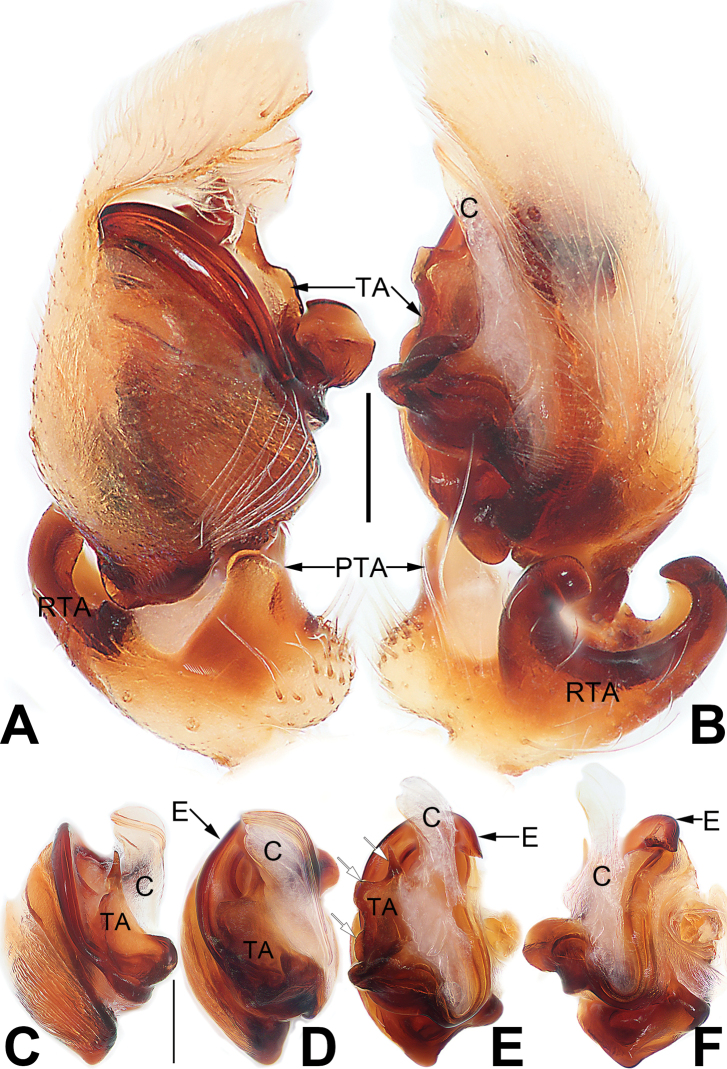
Male palp of the holotype of *Matidiaxieqian* sp. nov., left palp and bulb (**A, B, E, F**) and flipped right bulb (**C, D**) **A** prolateral view **B** retrolateral view **C** bulb, prolateral view **D** bulb, ventral view **E** bulb, ventrolateral view **F** retrolateral view. Arrows (**E**) point at three processes of tegular apophysis. Abbreviations: C = conductor; E = embolus; PTA = prolateral tibial apophysis; RTA = retrolateral tibial apophysis; TA = tegular apophysis. Scale bars: 0.20 mm (equal for **A, B**, equal for **C–F**).

**Figure 6.**
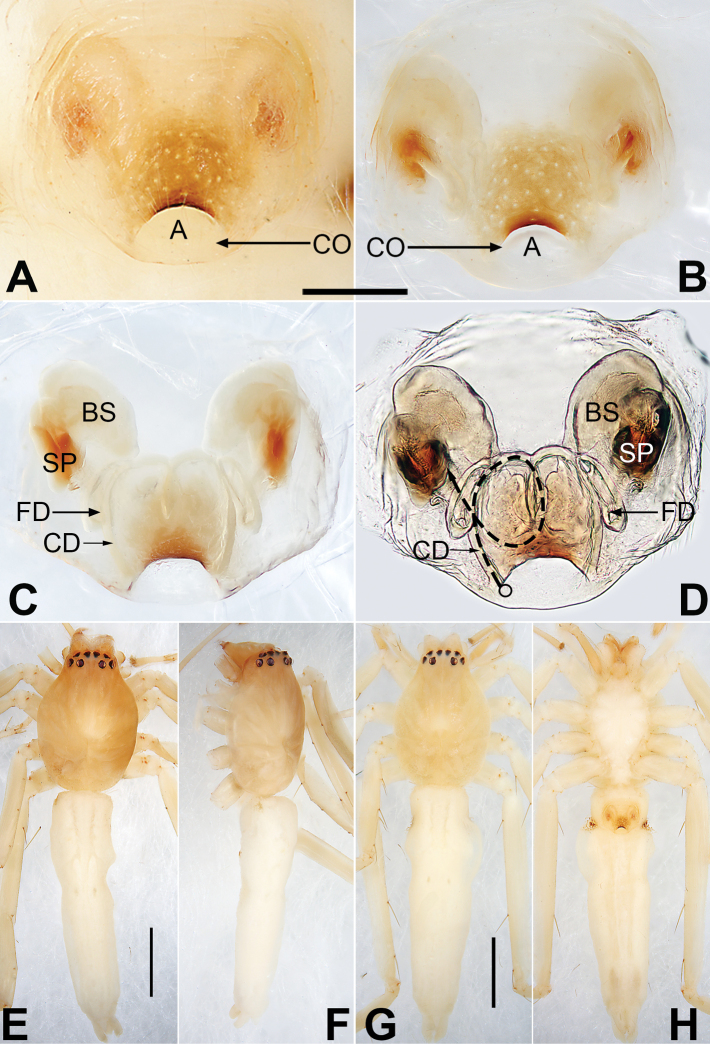
*Matidiaxieqian* sp. nov., female paratype and male holotype, epigyne (**A–D**), male habitus (**E, F**) and female habitus (**G, H**) **A** intact, ventral view **B** cleared, ventral view **C** cleared, dorsal view **D** cleared, dorsal view; path of copulatory duct marked **E** dorsal view **F** lateral view **G** dorsal view **H** ventral view. Abbreviations: A = epigynal atrium; BS = bursa; CD = copulatory duct (dashed line showing schematic course of copulatory duct, dorsal); CO = copulatory opening; FD = fertilization duct; SP = spermatheca. Scale bars: 0.20 mm (equal for **A–D**); 1 mm (equal for **E, F**, equal for **G, H**).

Species in this genus differ from all other clubionids by the following: the pars cephalica is 2× narrower than the pars thoracica (Figs 4E, G, 6E–G) (vs. wider), leg I is longest (vs. not longest), and there is a dark ventral abdominal spot in males (Fig. 4F) (vs. absent). *Matidia* resembles *Malamatidia*, *Nusatidia*, and *Ramosatidia* gen. nov. by the slender, greenish body but is consistently separable by the shape of the copulatory organs: the male palp has a ribbon-shaped embolus (Figs 3A, C–F, 5A, C–F) (vs. embolus not ribbon-shaped), the epigyne has one or two depressions (or an atrium) and no septum (Figs 4A, B, 6A, B) (vs. depression lacking in *Nusatidia* and *Ramosatidia* gen. nov., or present but with a septum in *Malamatidia*).

##### Comments.

Based on the two newly discovered species, the description should be extended from Deeleman-Reinhold (2001): the epigyne of *M.spatulata* Chen & Huang, 2006 with 2 depressions, copulatory ducts relatively long (longer than epigyne) (Fig. 4A–D) (vs. one central atrium and short ducts (shorter than epigyne) in all other congeners (e.g., *M.xieqian* sp. nov.; Fig. 6A–D)); an additional exceptional feature in *M.xieqian* sp. nov. is that the cheliceral promarginal teeth are farther from the fang base than the retromarginal ones (vs. promarginal teeth nearer the fang base in all other known *Matidia* species). Both *M.spatulata* and *M.xieqian* sp. nov. have anteriorly located bursae (vs. bursae located laterally or posteriorly).

##### Note.

Deeleman-Reinhold (2001) considered *Matidia* putatively polyphyletic. In the same work, she established three new, similar closely related genera (*Nusatidia*, *Pristidia*, and *Malamatidia*) to accommodate 12 new species from SE Asia, and placed them in the subfamily Clubioninae. She also transferred four *Matidia* species to *Nusatidia*. The similar somatic characters and sympatric distribution strongly suggest close relationships between the four genera. However, the phylogenetic relationships remain unresolved (Versteirt et al. 2010).

#### Matidia spatulata

Taxon classificationAnimaliaAraneaeClubionidae

Chen & Huang, 2006

737F9740-C401-5C11-A48F-183A15DAB80F

Figs 3 , 4

Matidia spatulata Chen & Huang, 2006: 68, fig. 1A–C (♂); Huang and Chen 2012a: 1, figs 1–4 (♀); Huang and Chen 2012b: 29, fig. 29A–C, pl. 8C–D (♂♀).

##### Material examined.

China: **Yunnan**: Xishuangbanna: Mengla County: Menglun Town: Menglun Nature Reserve: 3♂, rubber plantation (21°54.350'N, 101°16.461'E, 610 m), 11 August 2007, G. Zheng leg.; 1♀, G213 roadside, low evergreen forest (21°53.794'N, 101°17.152'E, 590 m), 27 November 2009, G. Tang eg.; 1♂1♀ (YHCLU0045–0046), secondary tropical forest (21°54.492'N, 101°16.866'E, 609 m), 31 July 2018, H. Yu leg.

##### Diagnosis and description.

See Huang and Chen (2012a, b). Male palp as in Fig. 3A–F, epigyne as in Fig. 4A–D, habitus as in Fig. 4E–H.

##### Distribution.

China (Taiwan, Yunnan Province).

#### Matidia xieqian

Taxon classificationAnimaliaAraneaeClubionidae

Yu & Li sp. nov.

6B51C26A-88B7-59F3-97FB-C556977F3155

http://zoobank.org/C948F60A-43D6-4898-BF0D-CD89EFF124A6

Figs 5 , 6

##### Type material.

***Holotype*** ♂ (IZCAS-Ar34728), China: **Yunnan**: Xishuangbanna: Mengla County: Menglun Town: Menglun Nature Reserve: secondary tropical montane evergreen broad-leaved forest (21°57.784'N, 101°11.947'E, 895 m), 6 August 2007, G. Zheng leg. ***Paratype***: 1♀ (IZCAS-Ar34729), secondary tropical montane evergreen broad-leaved forest (21°57.534'N, 101°12.300'E, 860 m), 4 August 2007, G. Zheng leg.

##### Other material examined.

China: **Yunnan**: Xishuangbanna, Mengla County: Menglun Town: Menglun Nature Reserve: 1♂ (YHCLU0126), 48 km landmark, seasonal rainforest (21°58.704'N, 101°19.748'E, 1088 m), 12 August 2011, G. Zheng leg.; Meng’a Town: 1♀ (YHCLU0127), Wengnan Village, secondary seasonal rain forest (22°05.002'N, 100°22.009'E, 1137 m), 30 June 2012, Q. Zhao leg.

##### Etymology.

The specific name is derived from the Chinese pinyin *xiè qián*, which means crab claw, referring to the concave retrolateral tibial apophysis with two branches resembling a crab claw; noun in apposition.

##### Diagnosis.

Males of *M.xieqian* sp. nov. can be distinguished from other *Matidia* species by the branched retrolateral tibial apophysis (Fig. 5B) and a tegular apophysis with three processes (Fig. 5E) (vs. RTA unbranched, tegular apophysis absent or present but with only process in other species). Females of this species resemble those of *M.simia* Deeleman-Reinhold, 2001 in having an epigynal atrium, lacking in all other *Matidia* species, but differ by the: (1) copulatory ducts 3× longer than spermathecae (Fig. 6C, D) (vs. < 2× longer than spermathecae); (2) spermathecae situated posteriorly to bursae (Fig. 6C, D) (vs. anteriorly); (3) spermathecae not coiled (Fig. 6C, D) (vs. coiled).

##### Description.

**Male** (holotype) (Fig. 6E, F). Total length 5.30; carapace 1.99 long, 1.50 wide; opisthosoma 3.32 long, 0.98 wide. Carapace yellowish brown posteriorly and centrally, dark anteriorly and marginally, without distinct pattern; cervical groove and radial groove distinct, fovea indistinct. Eyes: AER slightly recurved, PER wider than AER, almost straight in dorsal view. AME dark, other eyes light, with black rings. Eye sizes and interdistances: AME 0.08, ALE 0.10, PME 0.10, PLE 0.10, AME–AME 0.05, AME–ALE 0.05, PME–PME 0.22, PME–PLE 0.06, MOQL 0.28, MOQA 0.24, MOQP 0.44. Chelicerae coloured as ocular region, with 3 promarginal and 2 retromarginal teeth. Labium and endites light brown. Sternum yellowish white. Legs uniformly yellowish orange. Leg measurements: I 12.71(3.35, 5.08, 2.93, 1.35), II 10.53 (3.02, 4.26, 2.27, 0.98), III 6.02 (1.75, 2.11, 1.67, 0.50), IV 11.02 (3.11, 3.64, 3.46, 0.81). Abdomen lanceolate, white, dorsum with a lengthwise, white heart mark reaching posterior half of abdomen; pair of muscle depressions on both sides of heart-shaped mark; venter, spinnerets yellowish white.

Palp (Fig. 5A–F): Tibia short, ~ 1/4–1/3 of cymbium length, with two apophyses: PTA subtriangular in prolateral view, with a bulky base and blunt tip, ~ 1/2 of tibia length; RTA approximately as long as tibia, heavily sclerotised, strongly expanded, directed antero-dorsally, concave with two branches, shaped like a crab claw, ventral branch thumb-shaped, dorsal branch finger-like. Bulb elongated, with distinct, sinuate sperm duct. Tegular apophysis well developed, > 2/3 of tegulum length, with three processes: apico-prolateral process sharp, tooth-shaped, meso-prolateral process blunt, trapezoidal, baso-prolateral process represented by a blunt flange. Embolus wide, approximately as long as tegulum, originating at ~ 8 o’clock, beak-shaped tip terminated at ~ 1 o’clock position. Conductor thick, membranous, ~ 1.1–1.2× longer than embolus, originating baso-retrolaterally from tegulum, spoon-shaped distally.

**Female** (paratype IZCAS-Ar34729): Total length 5.46; carapace 2.10 long, 1.47 wide; opisthosoma 3.41 long, 0.98 wide. General characters as in male (Fig. 6G, H). Eye sizes and interdistances: AME 0.07, ALE 0.10, PME 0.11, PLE 0.09, AME–AME 0.06, AME–ALE 0.05, PME–PME 0.22, PME–PLE 0.05, MOQL 0.53, MOQA 0.21, MOQP 0.42. Legs white, without distinct markings. Leg measurements: I 10.10 (2.82, 4.15, 2.26, 0.87), II 6.59 (1.87, 2.37, 1.60, 0.75), III 5.62 (1.75, 1.89, 1.54, 0.45), IV 9.92 (3.05, 3.18, 2.98, 0.70).

Epigyne (Fig. 6A–D): Plate fan-shaped, length subequal to width, margins distinctly delimited, spermathecae and bursae indistinct through integument. Atrium large, ~ 1/4 of epigyne length and 1/3 of epigyne width, located at posterior portion of epigynal plate. Copulatory openings indistinct, located at basolateral atrial borders. Hyaline copulatory ducts ascending anteriorly, following atrial borders, curved at half their length to form a loop, and ascending to spermathecae. Spermathecae bean-shaped, ~ 1.5× longer than wide, at lateral portion of vulva, separated by three diameters. Fertilisation ducts as long as spermathecae, blade-shaped. Bursae hyaline, oval, much larger than spermathecae, ~ 1.5× longer than wide, situated anteriorly, separated by ~ 0.8 diameters, surface semi-transparent.

##### Distribution.

Known only from the type locality.

#### Genus Nusatidia

Taxon classificationAnimaliaAraneaeClubionidae

Deeleman-Reinhold, 2001

B7DDA492-3D6B-579E-9DD9-07A1B0729B8C

Nusatidia Deeleman-Reinhold, 2001: 166.

##### Type species.

*Matidiajavana* Simon, 1897 from Java, Krakatau.

##### Diagnosis.

**Figure 7.**
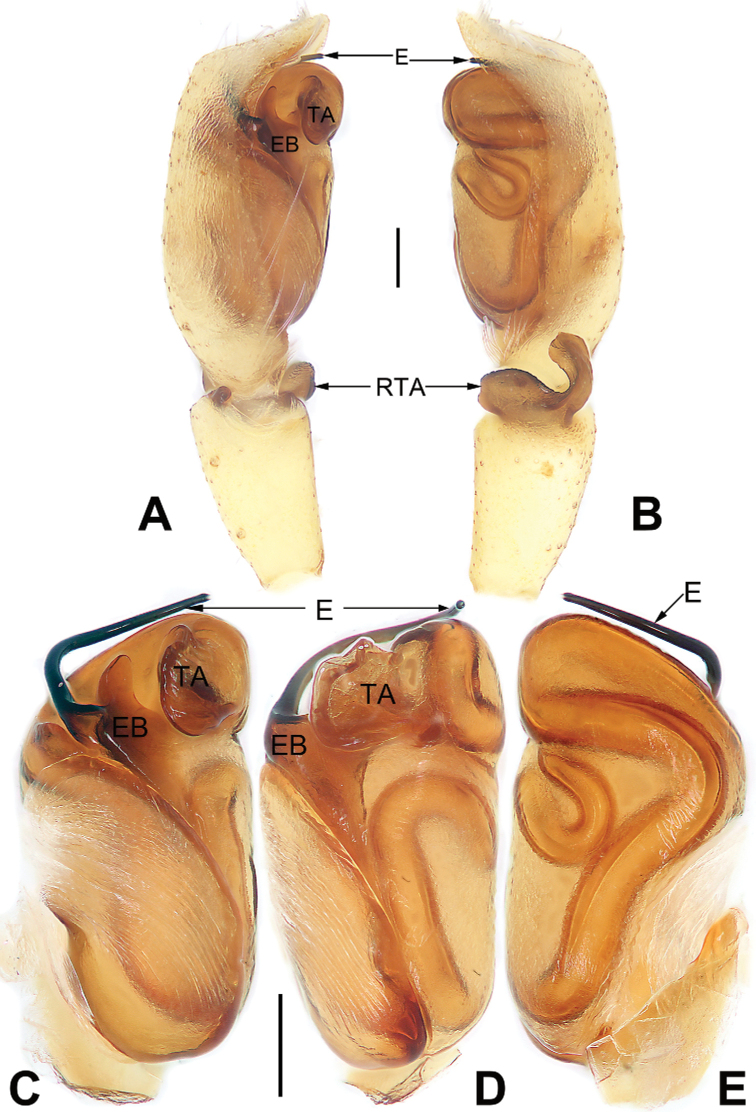
Male palp of *Nusatidiaaeria***A** prolateral view **B** retrolateral view **C** bulb, prolateral view **D** bulb, ventral view **E** bulb, retrolateral view. Abbreviations: E = embolus; EB = embolic base; RTA = retrolateral tibial apophysis; TA = tegular apophysis. Scale bars: 0.10 mm (equal for **A, B**, equal for **C–E**).

**Figure 8.**
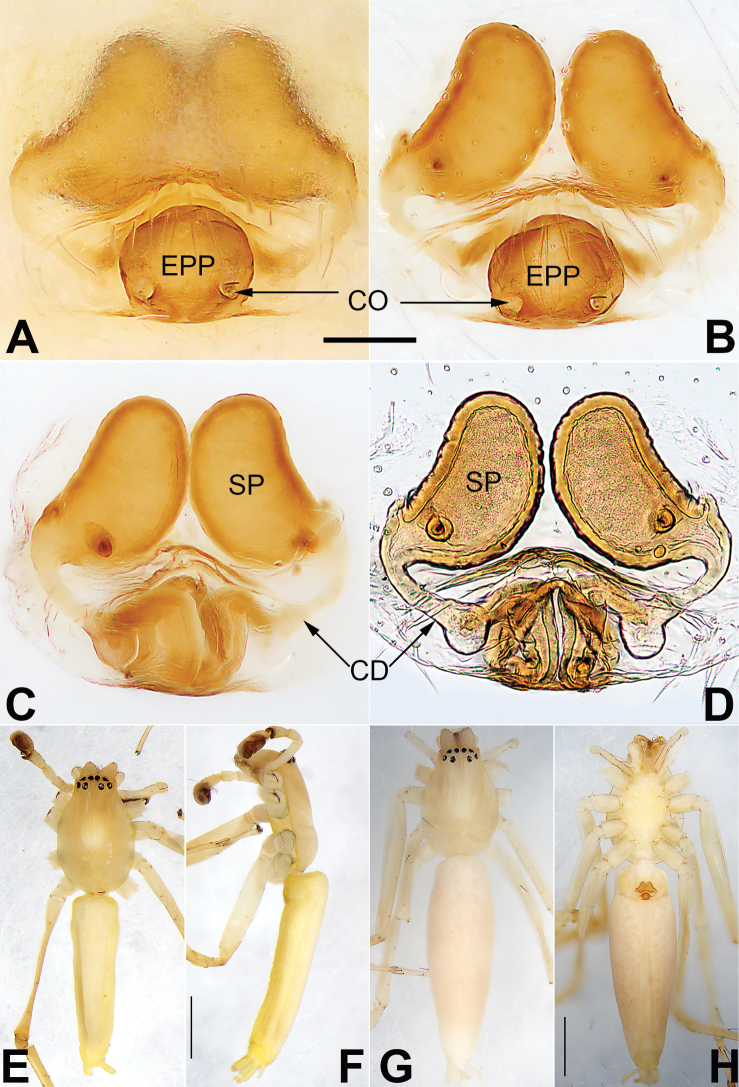
*Nusatidiaaeria*, epigyne (**A–D**), male habitus (**E, F**) and female habitus (**G, H**) **A** intact, ventral view **B** cleared, ventral view **C** cleared, dorsal view **D** cleared, dorsal view **E** dorsal view **F** lateral view **G** dorsal view **H** ventral view. Abbreviations: CD = copulatory duct; CO = copulatory opening; EPP = epigynal posterior plate; SP = spermatheca. Scale bars: 0.10 mm (equal for **A–D**); 1 mm (equal for **E, F**, equal for **G, H**).

**Figure 9.**
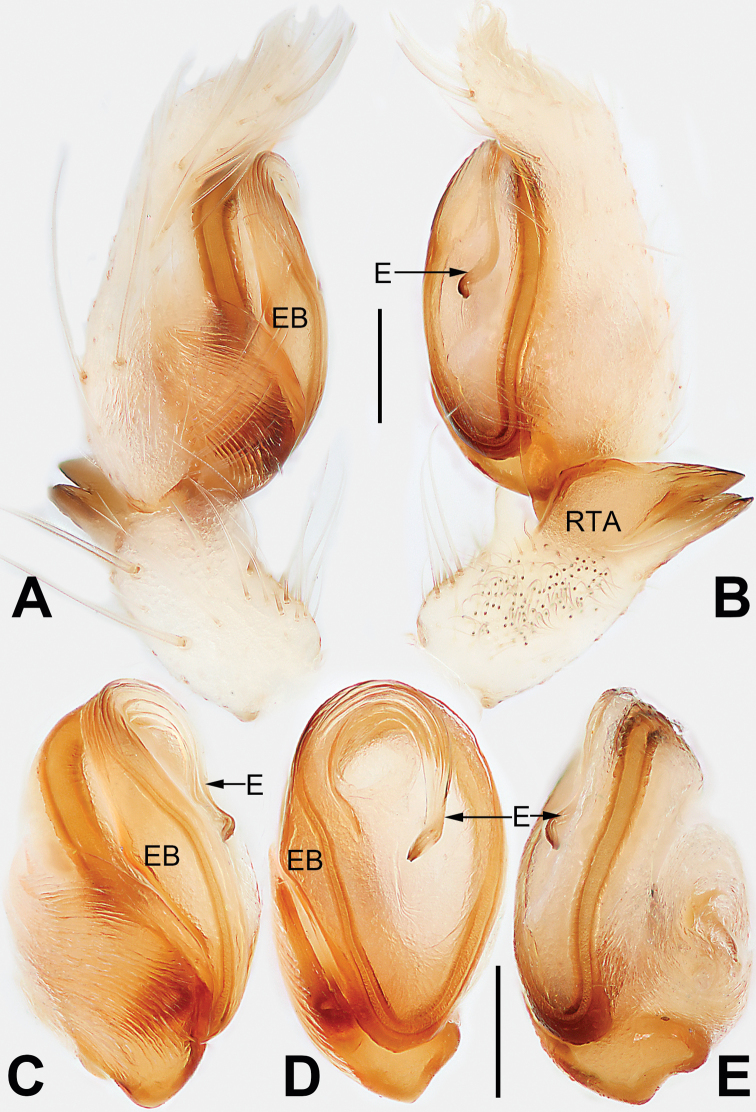
Male palp of *Nusatidiacamouflata***A** prolateral view **B** retrolateral view **C** bulb, prolateral view **D** bulb, ventral view **E** bulb, retrolateral view. Abbreviations: E = embolus; EB = embolic base; RTA = retrolateral tibial apophysis. Scale bars: 0.10 mm (equal for **A, B**, equal for **C–E**).

**Figure 10.**
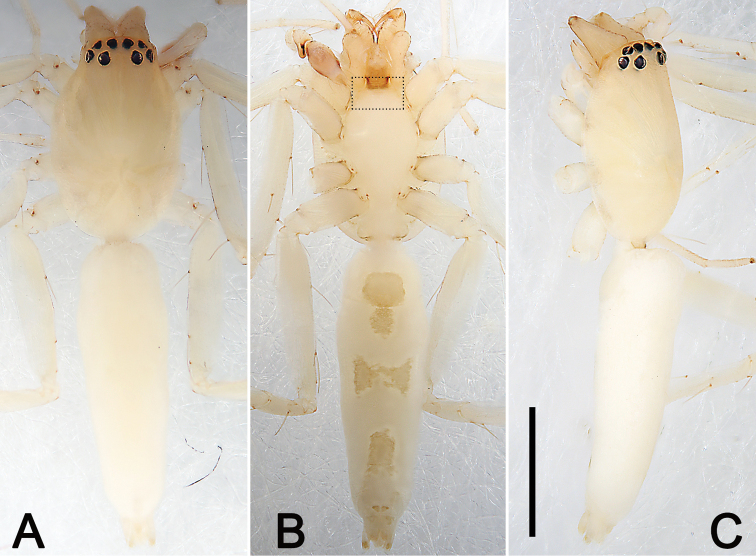
Male habitus of *Nusatidiacamouflata***A** dorsal view **B** ventral view **C** lateral view. Dotted box (B) showing rectangular projection in front of coxae I. Scale bars: 1 mm (equal for **A–C**).

**Figure 11.**
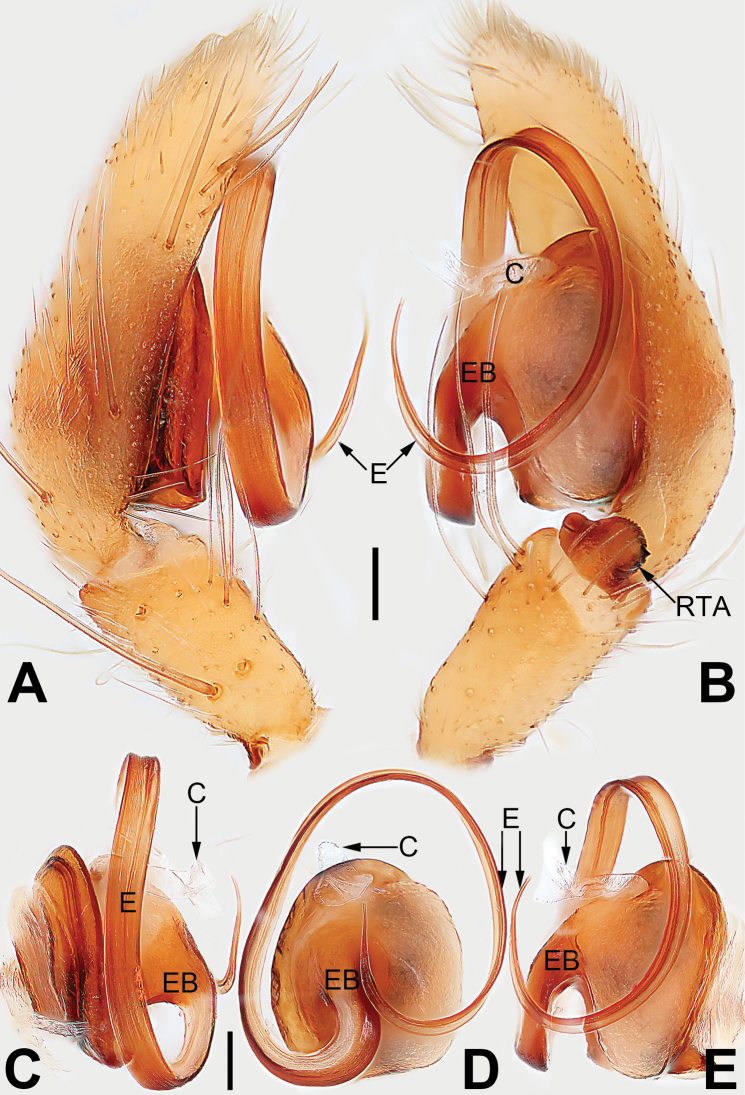
Male palp of the holotype of *Nusatidiachangao* sp. nov., left palp **(A, B)** and flipped right bulb (**C–E**) **A** prolateral view **B** retrolateral view **C** bulb, prolateral view **D** bulb, ventral view **E** bulb, retrolateral view. Abbreviations: C = conductor; E = embolus; EB = embolic base; RTA = retrolateral tibial apophysis. Scale bars: 0.10 mm (equal for **A, B**, equal for **C–E**).

**Figure 12.**
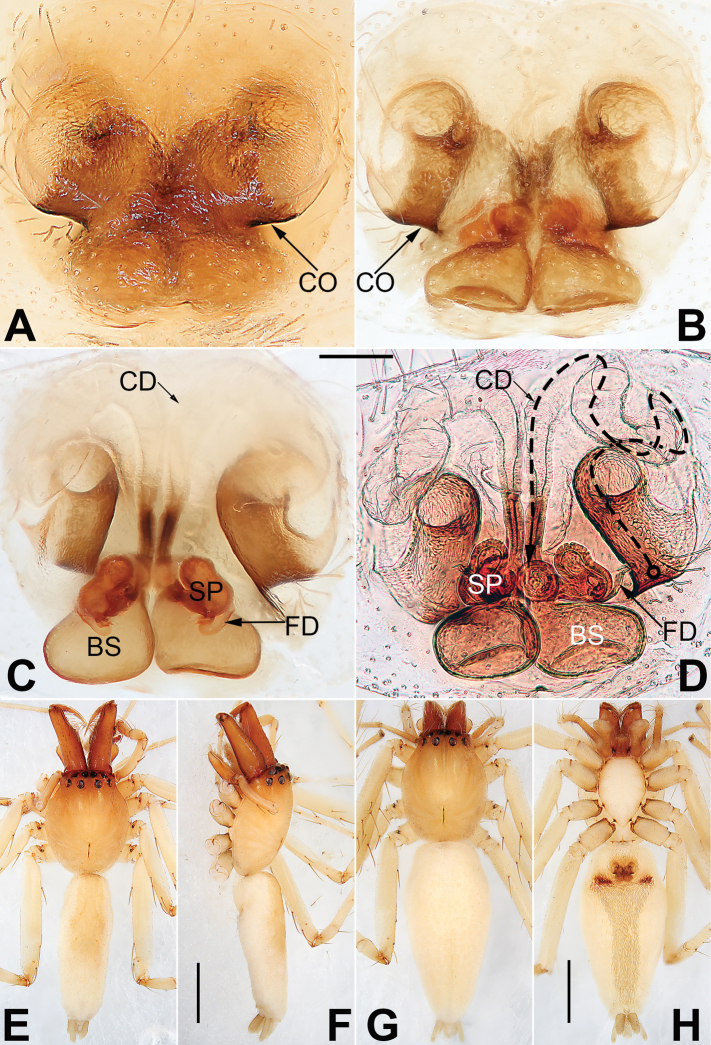
*Nusatidiachangao* sp. nov., female paratype and male holotype, epigyne (**A–D**), male habitus (**E, F**) and female habitus (**G, H**) **A** intact, ventral view **B** cleared, ventral view **C** cleared, dorsal view **D** cleared, dorsal view; path of copulatory duct marked **E** dorsal view **F** lateral view **G** dorsal view **H** ventral view. Abbreviations: BS = bursa; CD = copulatory duct (dashed line showing schematic course of copulatory duct, dorsal); CO = copulatory opening; FD = fertilization duct; SP = spermatheca. Scale bars: 0.10 mm (equal for **A–D**); 1 mm (equal for **E, F**, equal for **G, H**).

**Figure 13.**
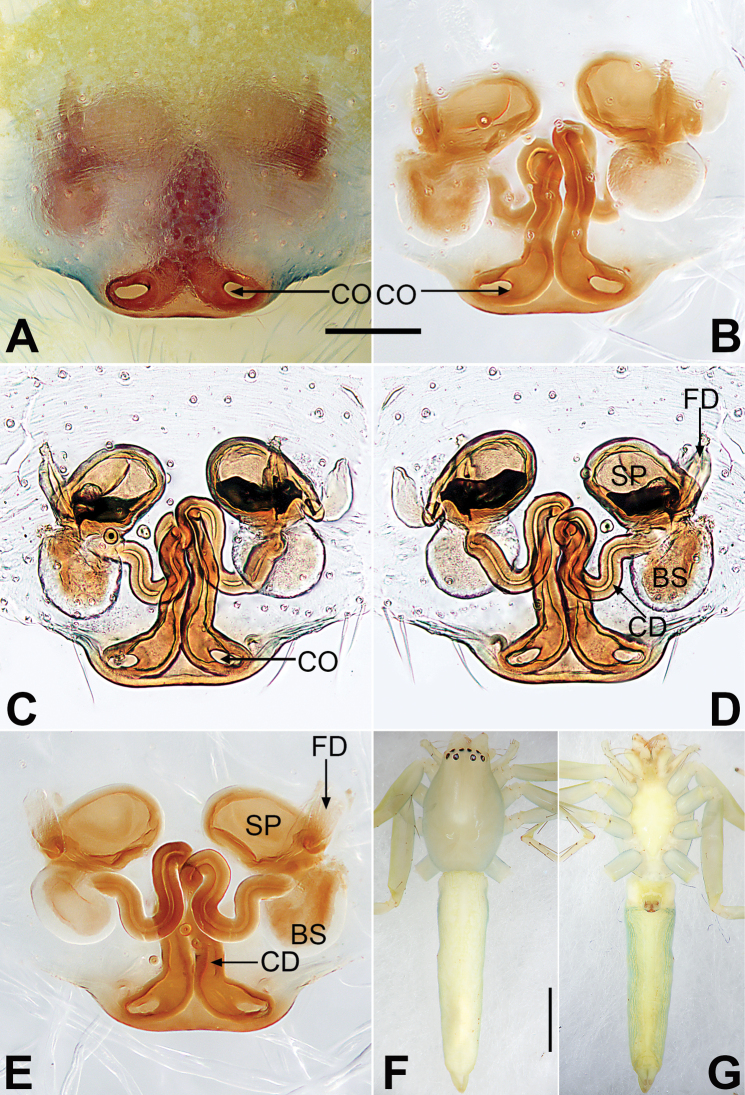
Holotype female of *Nusatidiamianju* sp. nov., epigyne (**A–E**) and habitus (**F, G**) **A** intact, ventral view **B** cleared, ventral view **C** cleared, ventral view **D** cleared, dorsal view **E** cleared, dorsal view **F** dorsal view **G** ventral view. Abbreviations: BS = bursa; CD = copulatory duct; CO = copulatory opening; FD = fertilization duct; SP = spermatheca. Scale bars: 0.10 mm (equal for **A–E**); 1 mm (equal for **F, G**).

**Figure 14.**
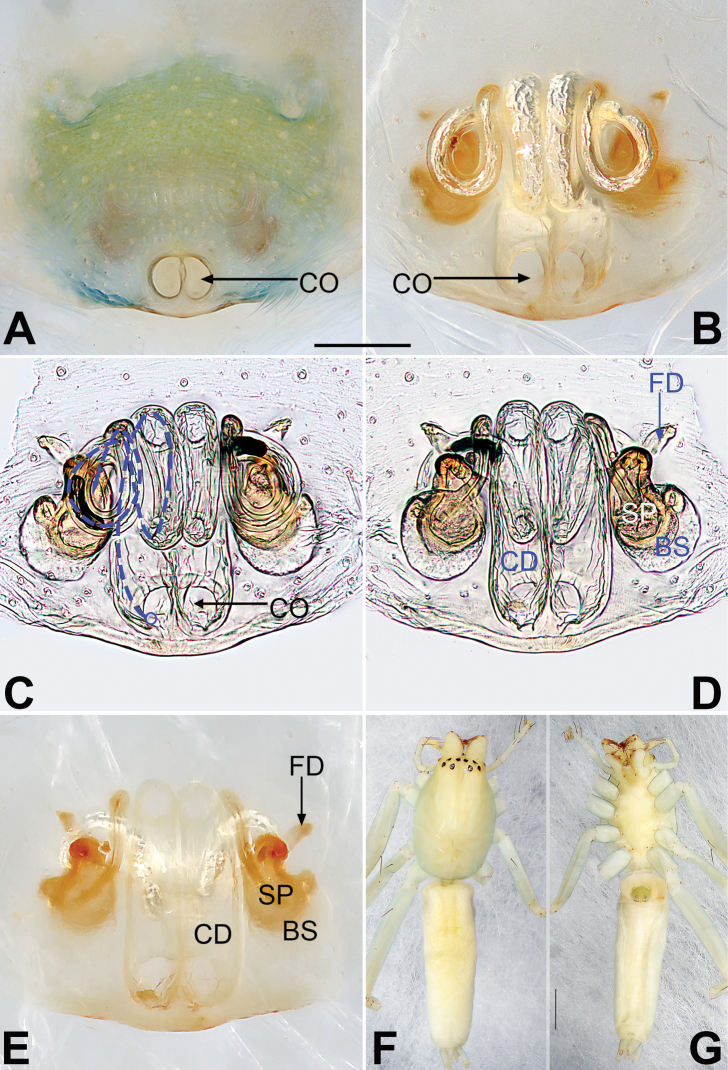
Holotype female of *Nusatidiasubjavana* sp. nov., epigyne (**A–E**) and habitus (**F, G**) **A** intact, ventral view **B** cleared, ventral view **C** cleared, ventral view; path of copulatory duct marked **D** cleared, dorsal view **E** cleared, dorsal view **F** dorsal view **G** ventral view. Abbreviations: BS = bursa; CD = copulatory duct (dashed line showing schematic course of copulatory duct, ventral); CO = copulatory opening; FD = fertilization duct; SP = spermatheca. Scale bars: 0.10 mm (equal for **A–E**); 1 mm (equal for **F, G**).

*Nusatidia* is very similar to *Malamatidia* and *Matidia* by the pale, slender body (Figs 2E–H, 4E–H, 8E–H), but it can be recognised by the following somatic characters: sternum with a rectangular projection beyond coxae I (Fig. 10B) (vs. sternum unmodified in all other SE Asian clubionids, such as *Malamatidia* and *Matidia* (Figs 2H, 4H)); leg I shorter than legs II and IV (vs. leg I longest in *Matidia*); male abdomen ventrally without pigmented spot (Fig. 10B) (vs. abdomen with dark spot on venter in *Matidia*; Fig. 4F). In general, most *Nusatidia* species can be recognised by the male palp: threadlike embolus draped around tegulum, such as in *N.camouflata* (Fig. 9A–E) and *N.changao* (Fig. 11A–E), but not in *N.luzonica* (Simon, 1897) and *N.aeria* (Simon, 1897) (Fig. 7A–E). Despite the variable general shape of the epigyne, all *Nusatidia* species lack a central depression (Figs 12A, B, 13A–C, 14 A–C) (vs. epigynal plate with a central depression in *Malamatidia* (Fig. 2A, B) or with one or two depressions in *Matidia* (Figs 4A, B and 6A, B)).

##### Description.

See Deeleman-Reinhold (2001).

##### Comments.

The somatic characters of *Nusatidia* species strongly suggest a close relationship with *Matidia*. However, both genera are possibly paraphyletic (Versteirt et al. 2010). According to WSC (2021), only two described *Nusatidia* species were known from males: *N.luzonica* (Simon, 1897) and *N.manipisea* (Barrion & Litsinger, 1995), both from Luzon Island in the Philippines. We cannot rule out the possibility that these two species are conspecific to *C.mianju* sp. nov. and *C.subjavana* sp. nov.

#### Nusatidia aeria

Taxon classificationAnimaliaAraneaeClubionidae

(Simon, 1897)

DA58D20D-18DE-5AC8-BBF6-D5FEDD88DA5B

Figs 7 , 8

Matidia aeria Simon, 1897: 50 (♀).
Nusatidia aeria : Deeleman-Reinhold 2001: 179, fig. 177 (♀, transferred to Nusatidia).
Nusatidia rama Deeleman Reinhold, 2001: 181, figs 178–180 (♂). syn. nov.

##### Material examined.

China: **Yunnan**: Xishuangbanna: Mengla County: Xishuangbanna Nature Reserve: 1♂1♀, Xiaolongha biodiversity preservation corridor (21°24.159'N, 101°37.178'E, 630 m), 27 June 2012, Q. Zhao leg.; 1♂ (YHCLU0150), Huigang Village, ecological restoration area of chevrotain, seasonal rainforest (21°37.045'N, 101°35.268'E, 760 m), 12 June 2012, Q. Zhao leg.; 1♀ (YHCLU0149), Nanshahe Village, seasonal rainforest (21°36.338'N, 101°34.247'E, 790 m), 13 June 2012, Q. Zhao leg.

##### Diagnosis.

Males of *N.aeria* are similar to those of *N.luzonica* by the elongate-oval bulb with a bulky, twisted sperm duct and the needle-shaped embolus (Fig. 7A–E), but they differ by the large and branched retrolateral tibial apophysis (Fig. 7B) (vs. RTA small, indistinct, and not branched) and by the presence of a petal-shaped tegular apophysis (Fig. 7A, C, D) (vs. tegular apophysis lacking). Females of *N.aeria* can be easily recognised by having a subcircular plate located at the posterior of the epigynal plate (Fig. 8A, B) (vs. posterior plate absent in all other *Nusatidia* species).

##### Description.

See Deeleman-Reinhold (2001). Male palp as in Fig. 7A–E, epigyne as in Fig. 8A–D, habitus as in Fig. 8E–H.

##### Comments.

*Nusatidiaaeria* was originally described in *Matidia* based on the holotype female from Jolo Island, Philippines. Deeleman-Reinhold (2001) examined the holotype and transferred the species to *Nusatidia*. In the same work, she described *N.rama* based on the holotype male from Sumatra but suggested that these two species could be conspecific. Recently, new material has been collected from Xishuangbanna containing both sexes. The males were identified as *N.rama* while the females were identified as *N.aeria*. On the basis of the morphological characters (Fig. 8E–H) and DNA barcoding, we matched the females and males. Therefore, the two species are synonymised, and priority is given to *N.aeria*.

##### Distribution.

Prior to our study, this species was known from Borneo and Indonesia (Sumatra) only. Our collection in southwest China (Yunnan Province, new record) extends the known range of this species ~ 2700 km to the northwest.

#### Nusatidia camouflata

Taxon classificationAnimaliaAraneaeClubionidae

Deeleman-Reinhold, 2001

A6DFCBA9-50B9-5CC0-B350-2FEA9FDC0242

Figs 9 , 10

Nusatidia camouflata Deeleman-Reinhold, 2001: 176, figs 169–174 (♂♀).

##### Material examined.

China: **Yunnan**: Xishuangbanna: Mengla County: Menglun Town: Menglun Nature Reserve: 1♂, secondary tropical montane evergreen broad-leaved forest (21°57.528'N, 101°12.384'E, 899 m), 4–11 May 2007, G. Zheng leg.

##### Diagnosis.

Males of this species can be easily distinguished from congeners by the long and bifid retrolateral tibial apophysis, over 1/2 of tibial length (Fig. 9B) (vs. RTA not bifid, < 1/3 of tibial length). The male also can be easily recognised by having abdominal patterns on venter (Fig. 10B) (vs. lacking pattern).

##### Description.

See Deeleman-Reinhold (2001). Male palp as in Fig. 9A–E, habitus as in Fig. 10A–C.

##### Distribution.

Prior to our study, this species was known from Thailand (Kanchanaburi Province) only. Our collection in southwest China (Yunnan Province, new record) extends the known range of this species ~ 870 km to the northwest.

#### Nusatidia changao

Taxon classificationAnimaliaAraneaeClubionidae

Yu & Li sp. nov.

2E83F654-A1C4-5ECB-AF2A-43AD16461D0A

http://zoobank.org/3051A41A-0FB2-4EBC-A194-7466527CF238

Figs 11 , 12

##### Type material.

***Holotype*** ♂ (IZCAS-Ar34731), China: **Yunnan**: Xishuangbanna: Mengla County: Menglun Town: Menglun Nature Reserve: Secondary tropical montane evergreen broad-leaved forest (21°57.528'N, 101°12.384'E, 899 m), 6 August 2007, G. Zheng leg. ***Paratype***: 1♀ (IZCAS-Ar34732), same data as holotype.

##### Other material examined.

China: **Yunnan**: Xishuangbanna: Mengla County: Menglun Town: Menglun Nature Reserve: 1♂ (YHCLU0152), 48 km landmark in Nature Reserve, seasonal rainforest (21°58.704'N, 101°19.748'E, 1080 m), 12 August 2011, G. Zheng leg.; Mengyang Town: Nabanhe Nature Reserve: 1♀ (YHCLU0129), waterfall, seasonal rainforest (22°7.607'N, 100°40.540'E, 730 m), 22 August 2012, G. Zheng leg.

##### Etymology.

The specific name is derived from the Chinese pinyin *cháng áo*, meaning long chelicerae, referring to the enlarged chelicerae of the male, which are approximately as long as the carapace; noun in apposition.

##### Diagnosis.

Males of *N.changao* sp. nov. resemble those of *N.borneensis* Deeleman-Reinhold, 2001 and *N.snazelli* Deeleman-Reinhold, 2001 in having a similar embolus draped around the tegulum but differ by the tibial apophysis, which has a flange with jagged teeth like those on a saw (Fig. 11B) (vs. smooth flange). Females of *N.changao* sp. nov. can be easily recognised by the lateral margins of the epigynal plate with copulatory openings under deep slits (Fig. 12A–D) (vs. epigynal plate without lateral slits, copulatory openings located posteriorly). Males of this new species also can be easily recognised by the enlarged chelicerae, ~ as long as the carapace (Fig. 12E) (vs. chelicerae unmodified, < 1/2 of carapace length).

##### Description.

**Male** (holotype) (Fig. 12E, F): Total length 3.98; carapace 1.70 long, 1.48 wide; opisthosoma 2.29 long, 0.92 wide. Carapace red wine coloured, pars cephalica darker in ocular area, without distinct pattern; ocular region distinctly narrowed; cervical groove indistinct; tegument smooth, with short setae. Eyes: AER almost straight, PER wider than AER and slightly procurved in dorsal view. AME dark, other eyes light; with black rings. Eye sizes and interdistances: AME 0.10, ALE 0.14, PME 0.14, PLE 0.11, AME–AME 0.04, AME–ALE 0.08, PME–PME 0.21, PME–PLE 0.12, MOQL 0.34, MOQA 0.28, MOQP 0.45. Chelicerae protruded, approximately equal in length to carapace, coloured as ocular area, with five promarginal and two retromarginal teeth. Labium and endites coloured as chelicerae. Sternum yellowish white. Legs white. Leg measurements: I 8.12 (2.23, 3.37, 1.72, 0.80), II 9.28 (2.56, 3.88, 1.99, 0.85), III 6.24 (1.81, 2.01, 1.79, 0.58), IV 8.93 (2.46, 2.96, 2.72, 0.79). Abdomen (Fig. 12E, F) dorsum yellowish white, dorsally with a wide scutum extended ~ 1/2 of abdomen length, gradually widened posteriorly, two pairs of inconspicuous sigilla on either side; venter, spinnerets light yellow.

Palp (Fig. 11A–E): Tibia relatively long, ~ 1/2 cymbium length; RTA stout, ~ 1/3–1/4 tibia length, with broad base and flange with jagged teeth like those on a saw. Bulb spherical, nearly as wide as long, sperm duct inconspicuous. Embolus at least 3× longer than tegulum, originating at centre of tegulum, draped around the tegulum, tapered to filiform, tip extended to anterior portion of tegulum, directed to 12 o’clock. Conductor large, membranous, at ~ 11 o’clock position.

**Female** (paratype IZCAS-Ar34732): Total length 4.89; carapace 1.93 long, 1.45 wide; opisthosoma 2.97 long, 1.46 wide. Similar to males but with distinctly smaller chelicerae and longer body (Fig. 12G, H). Eye sizes and interdistances: AME 0.08, ALE 0.09, PME 0.11, PLE 0.12, AME–AME 0.04, AME–ALE 0.06, PME–PME 0.18, PME–PLE 0.12, MOQL 0.30, MOQA 0.25, MOQP 0.41. Leg measurements: I 6.45 (1.89, 2.63, 1.26, 0.68), II 6.73 (1.92, 2.80, 1.34, 0.68), III 5.09 (1.62, 1.51, 1.43, 0.53), IV 7.28 (2.04, 2.25, 2.30, 0.69).

Epigyne (Fig. 12A–D): Plate trapezoidal, broad, nearly as wide as long, lateral margins concave medially, forming 2 windows. Copulatory openings inconspicuous, located at windows. Hyaline copulatory ducts long, strongly convoluted, proximally enlarged, cup-shaped, ducts ascending obliquely to middle, expanded laterally, then retracing anteriorly to form oblique arch, descending posteriorly to spermathecae. Spermathecae peanut-shaped, centrally located, separated by 1.5 diameters of a spermatheca. Bursae oblong, hyaline, situated posteriorly, close together, ~ 1.5× wider than long, surface translucent, smooth. Fertilisation ducts acicular, originating on posterior surface of spermathecae.

##### Distribution.

Known only from the type locality.

#### Nusatidia mianju

Taxon classificationAnimaliaAraneaeClubionidae

Yu & Li sp. nov.

B328E55E-0B09-5B9F-9B58-917BD4F7AD17

http://zoobank.org/02F77C69-57C8-463D-9828-C4B2A7E39AF7

Fig. 13

##### Type material.

***Holotype*** ♀ (IZCAS-Ar34730), China: **Yunnan**: Mengla County: Menglun Town: Menglun Nature Reserve: 48 km landmark in Nature Reserve, seasonal rainforest (21°58.704'N, 101°19.748'E, 1080 m), 12 August 2011, G. Zheng leg.

##### Other material examined.

China: **Yunnan**: Xishuangbanna: Mengla County: Meng’a Town: 1♀ (YHCLU0131), Wengnan Village, secondary seasonal rain forest (22°04.985'N, 100°22.217'E, 1130 m), 25 June 2012, Q. Zhao leg.

##### Etymology.

The specific name is derived from the Chinese pinyin *miàn jù*, for mask, referring to the conspicuousness of the spermathecae and copulatory ducts through the epigynal plate, the general appearance of a mask; noun in apposition.

##### Diagnosis.

Females of *N.mianju* sp. nov. are similar to those of *N.melanobursa* Deeleman-Reinhold, 2001 by the epigynal plate with a heavily sclerotised and convex posterior margin, and by the similar course of the copulatory ducts, but they can be differentiated by the copulatory openings separated by ~ 2 diameters (Fig. 13A, B) (vs. copulatory openings close together). *Nusatidiamianju* sp. nov. also resembles *N.camouflata* by the copulatory openings located at the posterolateral margin of the epigynal plate, separated by > 1.5 diameters, but it can be easily distinguished by having the copulatory ducts close together and ascending longitudinally (vs. copulatory ducts separated by > one diameter, running horizontally).

##### Description.

**Female** (holotype) (Fig. 13F–G): Total length 6.85; carapace 2.63 long, 1.67 wide; opisthosoma 4.22 long, 0.98 wide. Carapace light green, slightly lighter in cephalic area, without distinct colour pattern; cephalic region slightly narrowed, cervical groove, radial grooves, fovea indistinct. Eyes: AER slightly recurved, PER wider than AER and slightly procurved when seen from above. AME dark, other eyes light; with black rings. Eye sizes and interdistances: AME 0.10, ALE 0.09, PME 0.10, PLE 0.09, AME–AME 0.06, AME–ALE 0.08, PME–PME 0.23, PME–PLE 0.09, MOQL 0.30, MOQA 0.27, MOQP 0.47. Chelicerae white, fang light red, both promargin and retromargin with two teeth. Labium and endites light green. Sternum yellowish green. Legs coloured as carapace, without distinct markings. Leg measurements: I (2.03, —, —, —), II 8.42 (2.57, 3.33, 1.74, 0.79), III 5.56 (1.59, 1.99, 1.51, 0.47), IV 8.92 (2.69, 2.84, 2.75, 0.65). Abdomen (Fig. 13F, G) lanceolate, dorsum uniformly yellowish green; laterally dark green, with numerous longitudinal muscle depressions; venter without pattern, yellowish green centrally, dark green marginally.

Epigyne (Fig. 13A–E): Plate slightly wider than long, posterior margin heavily sclerotised and convex; spermathecae and copulatory ducts prominently visible through integument; in general appearance, epigynal plate like a mask. Copulatory openings distinct, large, separated by ~ 2 diameters, located at posterolateral margin of the plate. Hyaline copulatory ducts thick, straight, close together, extended to posterior level of spermathecae, then retracing posteriorly to mid-level of vulva, connected laterally to bursae. Spermathecae oval, ~ 1.4× longer than wide, situated anteriorly, separated by ~ 2/3 width of one spermatheca. Bursae globular, separated by ~ 1.3 diameters, translucent with smooth surface. Fertilisation ducts acicular, ~ 1/2 spermatheca length, located laterally.

**Male.** Unknown.

##### Distribution.

Known only from the type locality.

#### Nusatidia subjavana

Taxon classificationAnimaliaAraneaeClubionidae

Yu & Li sp. nov.

01F9F907-5D72-5118-A9B5-7FDD2B8C0F0A

http://zoobank.org/783EEDCD-34D7-4EB6-BA29-2E3A41A8577A

Fig. 14

##### Type material.

***Holotype*** ♀ (IZCAS-Ar34733), China: **Yunnan**: Xishuangbanna: Mengla County: Menglun Town: Menglun Nature Reserve: 48 km landmark in Nature Reserve, seasonal rainforest (21°58.704'N, 101°19.748'E, 1080 m), 12 August 2011, G. Zheng leg.

##### Other material examined.

1♀ (YHCLU0132), same data as holotype.

##### Etymology.

The specific name is a Latin adjective referring to the species’ similarity to *N.javana* (Simon, 1897), a combination of the preposition sub (near) and the epithet of that species.

##### Diagnosis.

Females of *N.subjavana* sp. nov. are similar to those of *N.javana* (Hayashi 1996: 66, figs 11–13) by the contiguous copulatory openings and the general shape of the vulva but can be distinguished from the latter by the strongly convoluted copulatory ducts that loop twice (Fig. 14B–D) (vs. moderately convoluted, loop once).

##### Description.

**Female** (holotype) (Fig. 14F–G): Total length 7.16; carapace 2.86 long, 1.96 wide; opisthosoma 4.30 long, 1.18 wide. Carapace uniformly green-white, lighter in cephalic region; radial grooves indistinct, fovea grey and inconspicuous. Eyes: AER nearly straight, PER wider than AER and slightly procurved in dorsal view. AME dark, other eyes light; with black rings. Eye sizes and interdistances: AME 0.09, ALE 0.11, PME 0.12, PLE 0.10, AME–AME 0.09, AME–ALE 0.11, PME–PME 0.32, PME–PLE 0.12, MOQL 0.34, MOQA 0.30, MOQP 0.55. Chelicerae protruding and coloured as carapace, with red wine coloured fangs, with three promarginal and two retromarginal teeth. Labium and endites white. Sternum white. Legs light green, without distinct markings. Leg measurements: I 9.23 (2.63, 4.00, 1.85, 0.78), II (—, —, 2.78, 0.79), III 6.12 (1.99, 2.09, 1.60, 0.44), IV (2.95, —, —, —). Abdomen (Fig. 14F, G): dorsum yellowish white with a pair of longitudinal muscle depressions, 1/3 of opisthosoma length; venter white, spinnerets light green.

Epigyne (Fig. 14A–E): Plate translucent greenish, slightly wider than long, through which part of spermathecae and copulatory ducts can be seen indistinctly, posterior margin bluish, not rebordered. Copulatory openings distinct, large, contiguous, at posterior portion of the plate. Copulatory ducts strongly entwined, loop twice before connecting to spermathecae. Sperm ~ 1.5× longer than wide. Bursae globular, separated by ~ 1.6 diameters, translucent, with smooth surface. Fertilisation ducts small, < 1/3 spermathecae length, located dorsolaterally on spermathecae.

**Male.** Unknown.

##### Distribution.

Known only from the type locality.

#### Genus Porrhoclubiona

Taxon classificationAnimaliaAraneaeClubionidae

Lohmander, 1944

674053A7-0F12-502F-91DE-A026DCF1CCAA

Porrhoclubiona Lohmander, 1944: 20; Prószyński and Staręga 1971: 234; Sterghiu 1985: 54 (considered a subgenus); Wunderlich 2011: 140 (considered a genus); Marusik and Omelko 2018: 22 (elevated to genus).
Clubiona : Mikhailov 2012: 179 (synonymised Porrhoclubiona Lohmander, 1944).
Clubiona genevensis -group: Bosmans et al. 2017: 2.
Clubiona pteronetoides -group: Deeleman-Reinhold 2001: 96.

##### Type species.

*Clubionagenevensis* L. Koch, 1866 from Switzerland.

##### Diagnosis.

**Figure 15.**
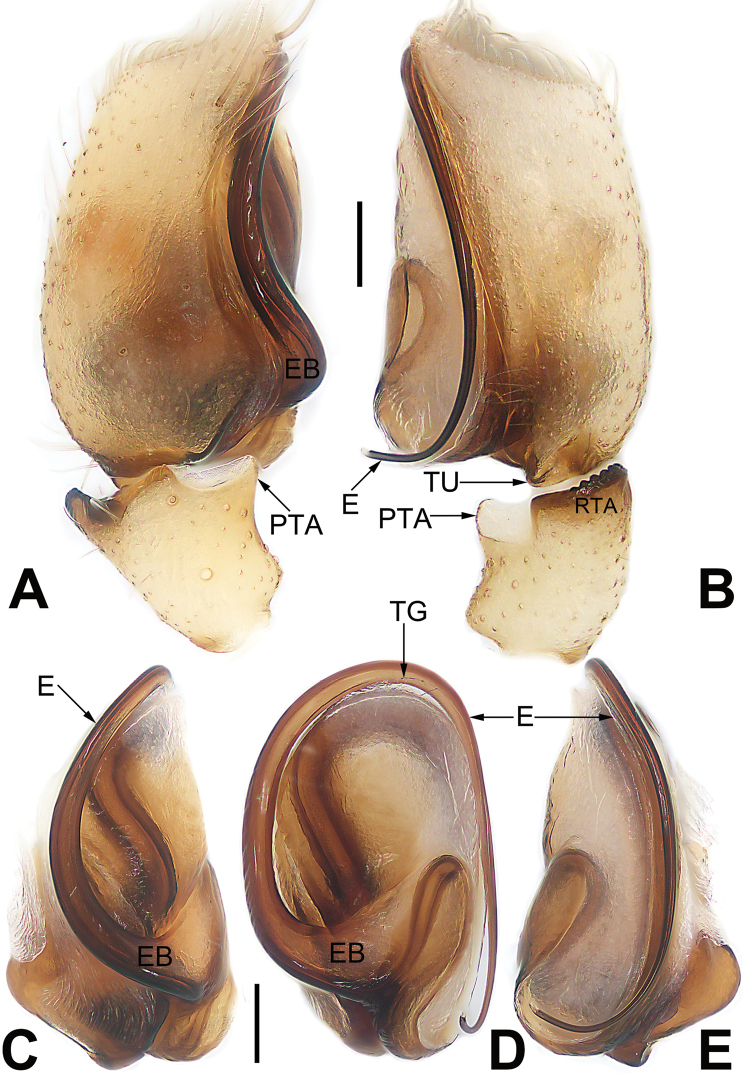
Male palp of *Porrhoclubionapteronetoides***A** prolateral view **B** retrolateral view **C** bulb, prolateral view **D** bulb, ventral view **E** bulb, retrolateral view. Abbreviations: E = embolus; EB = embolic base; RTA = retrolateral tibial apophysis; TG = tegular groove; TU = tutaculum. Scale bars: 0.10 mm (equal for **A, B**, equal for **C–E**).

**Figure 16.**
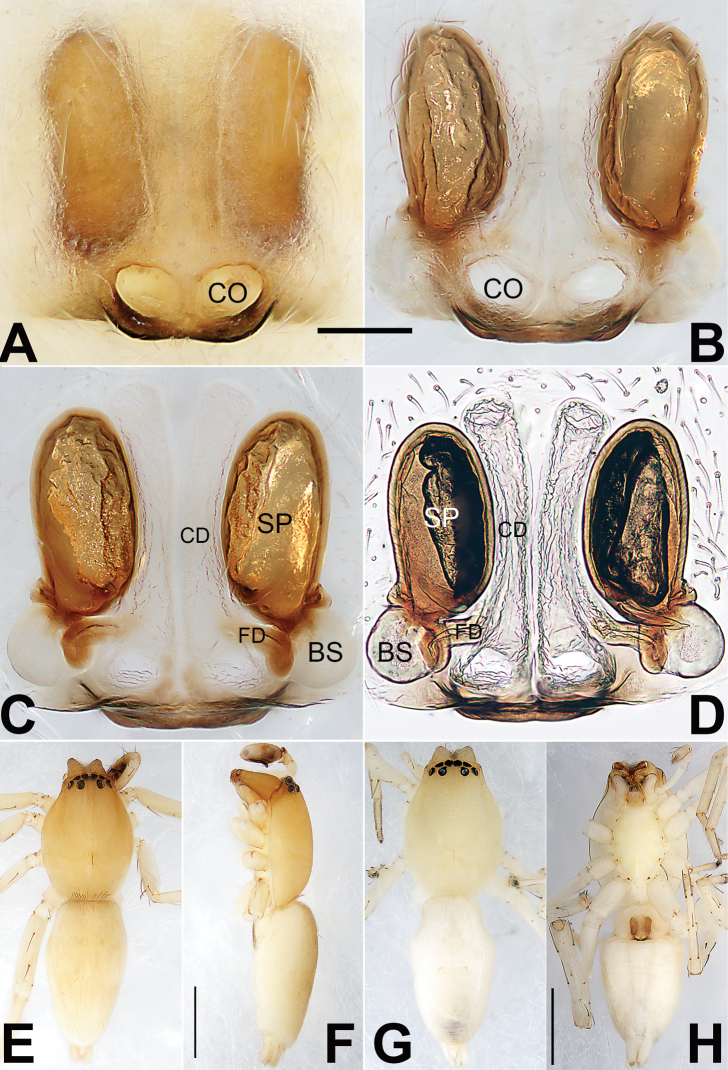
*Porrhoclubionapteronetoides*, epigyne (**A–D**), male habitus (**E, F**) and female habitus (**G, H**) **A** intact, ventral view **B** cleared, ventral view **C** cleared, dorsal view **D** cleared, dorsal view **E** dorsal view **F** lateral view **G** dorsal view **H** ventral view. Abbreviations: BS = bursa; CD = copulatory duct; CO = copulatory opening; FD = fertilization duct; SP = spermatheca. Scale bars: 0.10 mm (equal for **A–D**); 1 mm (equal for **E, F**, equal for **G, H**).

Distinct from all other clubionids by the cymbium with modified setae retrolaterally (not observed in *P.pteronetoides* Deeleman-Reinhold, 2001 Fig. 15B); cymbial base with a tutaculum (Fig. 15B); tegulum with a tegular groove serving as a conductor (Fig. 15D); tibia with a strongly reduced prolateral apophysis (Fig. 15A); subtegulum located posteriorly (Fig. 15C, D); sperm duct U-shaped, with an additional loop located proximally to embolic base (Fig. 15D); copulatory openings large, located posteriorly on ventral epigynal plate, united at midline, or separated but close (Fig. 16A, B); epigynal plate with a somewhat protruded posterior margin (Fig. 16A, B).

##### Description.

See Marusik and Omelko (2018) and Deeleman-Reinhold (2001).

##### Comments.

*Porrhoclubiona* is small Clubionidae with a relatively wide body and can be easily separated from *Malamatidia*, *Matidia*, and *Nusatidia. Porrhoclubiona* currently comprises two species groups, the *pteronetoides* group and the *genevensis* group. The *pteronetoides* group was established by Deeleman-Reinhold (2001) based on two species endemic to the Oriental realm. The *genevensis* group was formally named by Mikhailov (1992), though in fact it was first recognised by Lohmander (1944) as a subgenus of *Microclubiona* (currently considered junior synonyms of *Clubiona*), and then refined by Bosmans et al. (2017) with eight species from West Palaearctic region. The two groups share almost all of the generic characters listed by Marusik and Omelko (2018) (see diagnosis above); however, *pteronetoides* group can be differed from the *genevensis* group by have a dorsal abdominal scutum (Fig. 16E, F) (vs. absent), lack modified setae on the cymbium (Fig. 15B) (vs. present) in males, and the spermathecae are elongate in females (Fig. 16C, D) (vs. round).

#### Porrhoclubiona pteronetoides

Taxon classificationAnimaliaAraneaeClubionidae

(Deeleman-Reinhold, 2001)

FA20C05E-8CE1-5B47-8FFC-664E35C7D472

Figs 15 , 16

Clubiona pteronetoides Deeleman-Reinhold, 2001: 97, figs 1–7 (♂♀).
Porrhoclubiona pteronetoides : Marusik and Omelko 2018: 24 (transferred to Porrhoclubiona).

##### Material examined.

China: **Yunnan**: Xishuangbanna: Mengla County: Menglun Town: Menglun Nature Reserve: 1♂1♀, 48 km landmark in Nature Reserve, seasonal rainforest (21°58.704'N, 101°19.748'E, 1080 m), 12 August 2011, G. Zheng leg.; 1♂1♀ (YHCLU0124–125), 55 km landmark in Nature Reserve, seasonal rainforest (21°57.953'N, 101°12.305'E, 780 m), 13 August 2011, G. Zheng leg.

##### Diagnosis.

Both sexes of *P.pteronetoides* are similar to those of *P.viridula* in having similar palps and epigynes but can be differentiated by the elongate-oval bulb in ventral view (Fig. 15B) (vs. pyriform or triangular) and by the oval spermathecae that are ~ 2× longer than wide (Fig. 16C, D) (vs. claviform spermathecae that are ~ 3–4× longer than wide).

##### Description.

See Deeleman-Reinhold (2001). Male palp as in Fig. 15A–E, epigyne as in Fig. 16A–D, habitus as in Fig. 16E–H.

##### Comments.

There is almost no difference between males from Xishuangbanna (Fig. 15A–E) and the holotype from Thailand (Deeleman-Reinhold 2001: 97, figs 5–7). However, some intraspecific variation is exhibited by females from the two localities, related to distance between the two copulatory openings. The copulatory openings are separated by ~ 1/4 of their diameter in material from Xishuangbanna (Fig. 16A–D) but not separated in the paratype (Deeleman-Reinhold 2001: 97, fig. 4).

##### Distribution.

Thailand (Prachuap Khiri Khan Province), China (Yunnan Province, new record). The present results extend the range of this species by ~ 1090 km to the northwest (Xishuangbanna) from the type locality (Prachuap Khiri Khan).

#### Genus Pteroneta

Taxon classificationAnimaliaAraneaeClubionidae

Deeleman-Reinhold, 2001

BD954725-1AA5-56E5-89C2-DB8D10F656B6

Pteroneta Deeleman-Reinhold, 2001: 145.

##### Type species.

*Pteronetasaltans* Deeleman-Reinhold, 2001.

##### Diagnosis.

The genus is characterised by: peculiar scopula (called a feathery flag in Deeleman-Reinhold (2001)) on nearly the entire prolateral surface of the lengthened tarsi II (Fig. 18G), the patterned body with lazulite-coloured blue spots on the abdomen and coxae (Fig. 18E–H), the widely separated AME and PME (Fig. 18E, G), and the enlarged chelicerae of the male (Fig. 18E).

##### Description.

See Deeleman-Reinhold (2001).

#### Pteroneta ultramarina

Taxon classificationAnimaliaAraneaeClubionidae

(Ono, 1989)

BF87E6B3-C70B-5DA7-BFE7-958FCE167A46

Figs 17 , 18

Clubiona ultramarina Ono, 1989: 156, figs 1–7 (♂♀).
Pteroneta ultramarina : Deeleman-Reinhold 2001: 145 (transferred to Pteroneta) ; Ono and Hayashi 2009: 546, figs 7–10, 169–171 (♂♀).

##### Material examined.

China: **Yunnan**: Xishuangbanna: Mengla County: 1♂1♀ (YHCLU0136–137), Nanshahe Village, seasonal rainforest (21°36.200'N, 101°34.385'E, 820 m), 14 June 2012, Q. Zhao leg.; 1♂2♀, Bubang Village (21°36.634'N, 101°34.900'E, 820 m), 10 June 2012, Q. Zhao leg.; Menglun Town: Menglun Nature Reserve: 1♂, Lvshilin Forest Park, limestone tropical seasonal rain forest (21°54.617'N, 101°16.843'E, 730 m), 7 August 2011, G. Zheng leg.

##### Diagnosis.

**Figure 17.**
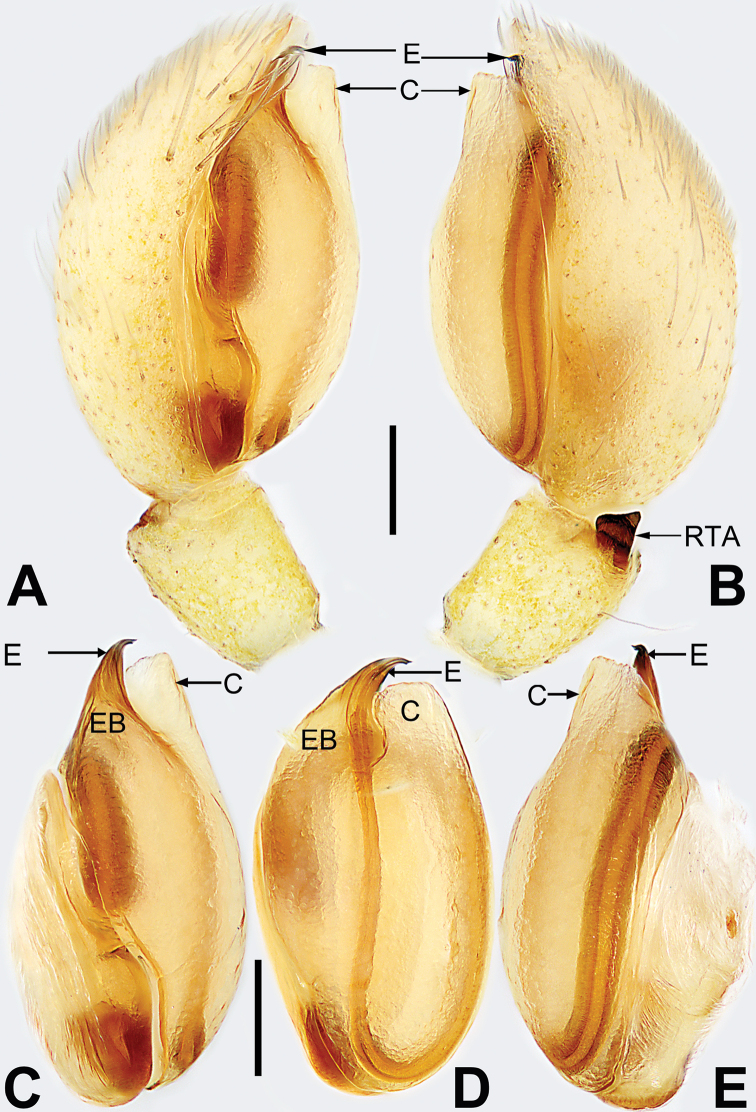
Male palp of *Pteronetaultramarina***A** prolateral view **B** retrolateral view **C** bulb, prolateral view **D** bulb, ventral view **E** bulb, retrolateral view. Abbreviations: C = conductor; E = embolus; EB = embolic base; RTA = retrolateral tibial apophysis. Scale bars: 0.10 mm (equal for **A, B**, equal for **C–E**).

**Figure 18.**
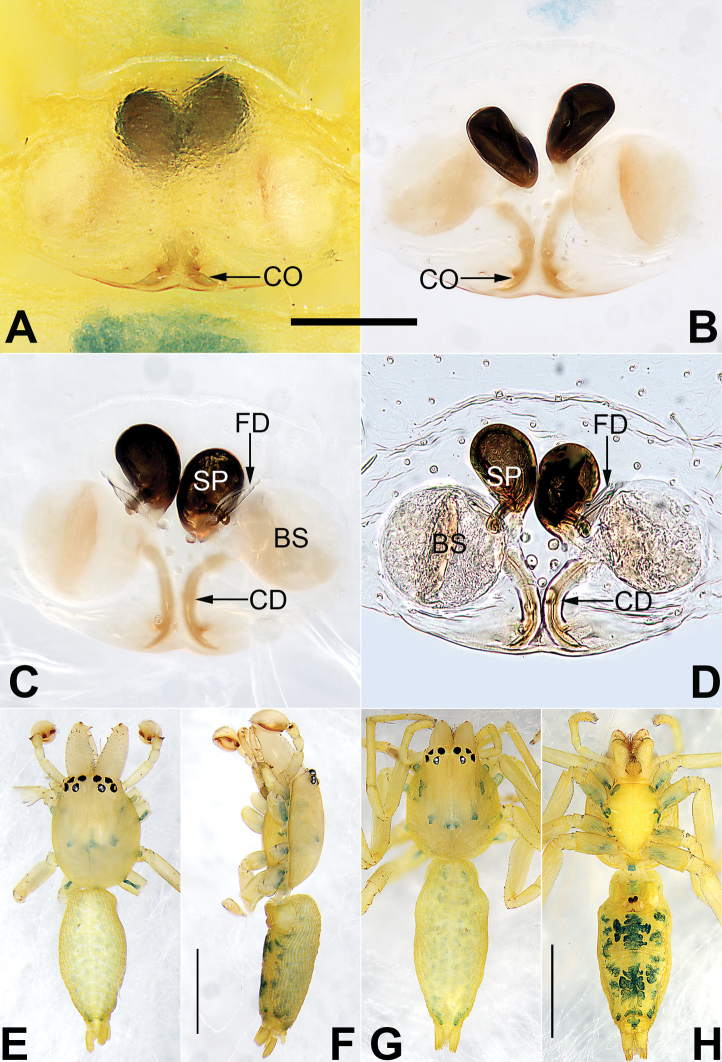
*Pteronetaultramarina*, epigyne (**A–D**), male habitus (**E, F**) and female habitus (**G, H**) **A** intact, ventral view **B** cleared, ventral view **C** cleared, dorsal view **D** cleared, dorsal view **E** dorsal view **F** lateral view **G** dorsal view **H** ventral view. Abbreviations: BS = bursa; CD = copulatory duct; CO = copulatory opening; FD = fertilization duct; SP = spermatheca. Scale bars: 0.10 mm (equal for **A–D**); 1 mm (equal for **E, F**, equal for **G, H**).

Males of *P.ultramarina* resemble those of *P.baiteta* Versteirt, Deeleman-Reinhold & Baert, 2008 (Versteirt et al. 2008: 312, fig. 7a, b) in having a similarly shaped retrolateral tibial apophysis and claw-like embolus but differ by the conductor with a straight tip (Fig. 17A–E) (vs. semi-circular tip) and the dorsal surface of the chelicerae with relatively few, short setae (< 10) (Fig. 18E) (vs. 18 short setae). Females of *P.ultramarina* are similar to those of *P.tertia* Deeleman-Reinhold, 2001. The epigyne of these two species is very similar and almost indistinguishable, but the species differ in the number and arrangement of cheliceral teeth (three promarginal and two retromarginal in *P.ultramarina* vs. six teeth on both margins in *P.tertia*) and by the patterns on the body (the carapace and sternum are marked with lazulite-coloured blue spots in *P.ultramarina* (Fig. 18G, H) (vs. blue spots lacking in *P.tertia*).

##### Description.

See Ono (1989). Male palp as in Fig. 17A–E, epigyne as in Fig. 18A–D, habitus as in Fig. 18E–H.

##### Distribution.

Japan (Ryukyu Is.), China (Yunnan Province, new record). The new record presented here extends the known range of this species by ~ 2700 km from the type locality (Ryukyu Is.) to the southwest (Xishuangbanna).

#### Ramosatidia

Taxon classificationAnimaliaAraneaeClubionidae

Yu & Li gen. nov.

BABA4A26-4DF5-5A86-8098-CFB55AE476D5

http://zoobank.org/0DA81522-8B4E-41B4-8325-03D62825768F

##### Type species.

*Ramosatidiasitu* Yu & Li, sp. nov.

##### Etymology.

The generic name is a combination of the Latin adjective *ramosus*, which means ramose, or branching, referring to the apophyses of the palpal tibia, in conjunction with *atidia*, alluding to the green colour and slender body, similar to *Matidia*. The gender is feminine.

##### Diagnosis.

**Figure 19.**
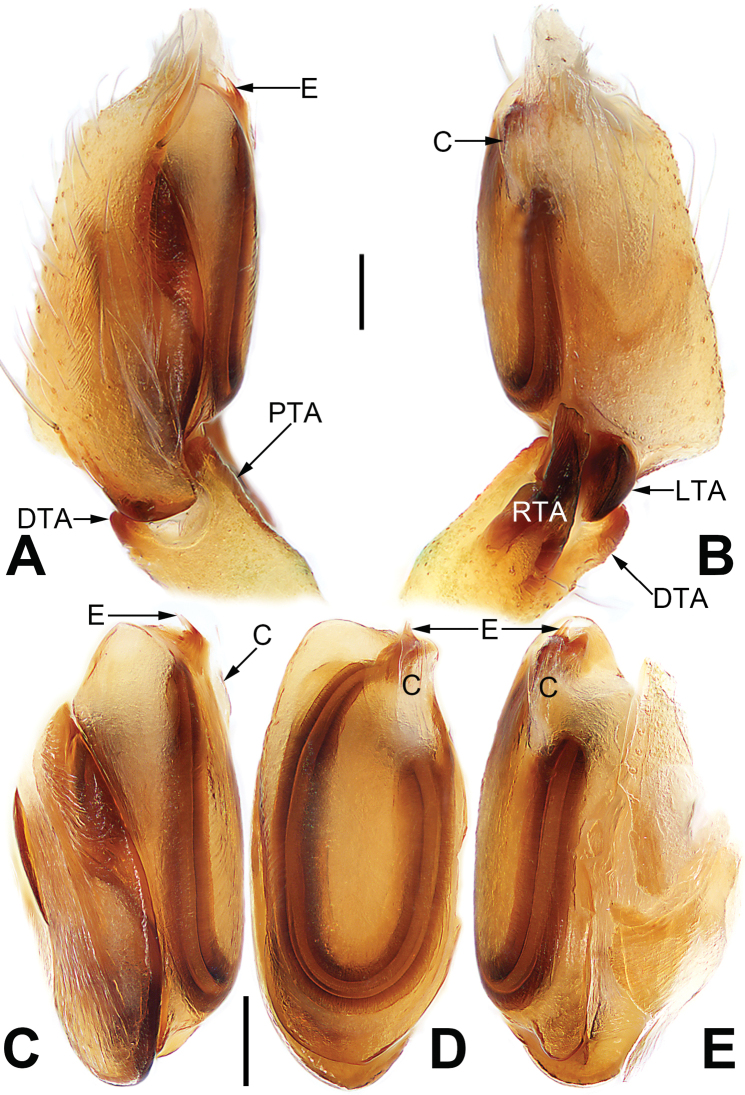
Male palp of the holotype of *Ramosatidiasitu* sp. nov. **A** prolateral view **B** retrolateral view **C** bulb, prolateral view **D** bulb, ventral view **E** bulb, retrolateral view. Abbreviations: C = conductor; DTA = dorsal tibial apophysis; E = embolus; LTA = lateral tibial apophysis; PTA = prolateral tibial apophysis; RTA = retrolateral tibial apophysis. Scale bars: 0.10 mm (equal for **A, B**, equal for **C–E**).

**Figure 20.**
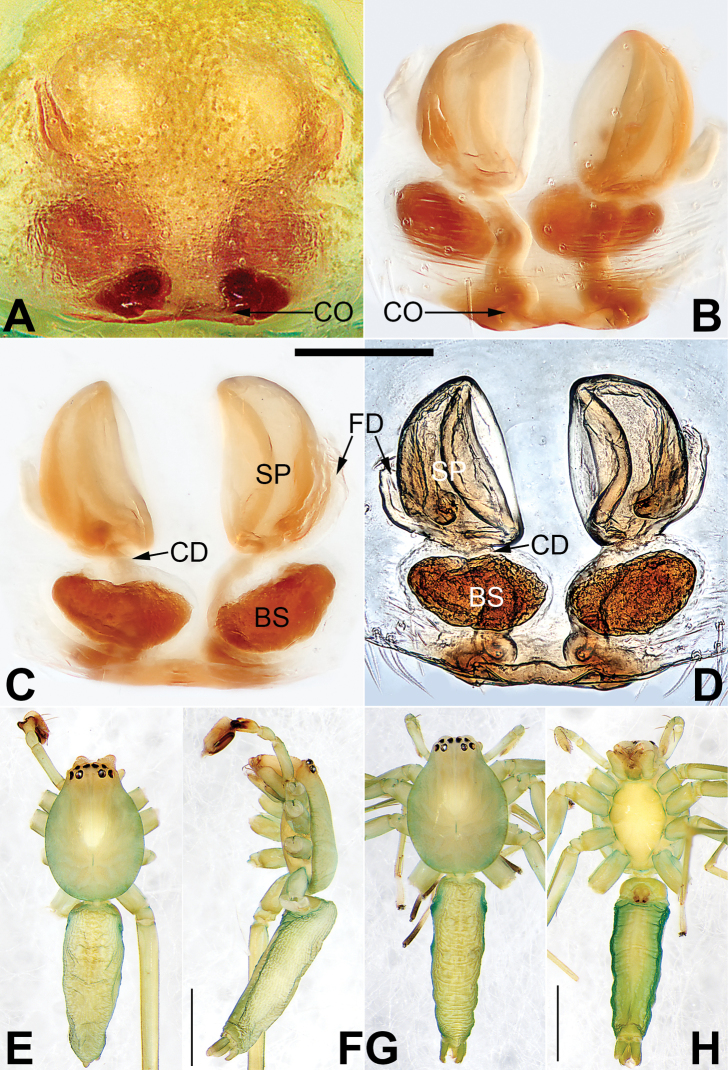
*Ramosatidiasitu* sp. nov., female paratype and male holotype, epigyne (**A–D**), male habitus (**E, F**) and female habitus (**G, H**) **A** intact, ventral view **B** cleared, ventral view **C** cleared, dorsal view **D** cleared, dorsal view **E** dorsal view **F** lateral view **G** dorsal view **H** ventral view. Abbreviations: BS = bursa; CD = copulatory duct; CO = copulatory opening; FD = fertilization duct; SP = spermatheca. Scale bars: 0.10 mm (equal for **A–D**); 1 mm (equal for **E, F**, equal for **G, H**).

*Ramosatidia* gen. nov. resembles the other genera exclusively distributed in SE Asia (*Pristidia*, *Nusatidia*, and *Matidia*) by the similar habitus (green, elongate, long-legged), but it is consistently separable by somatic characters and the copulatory organs. This new genus is characterised by the promargin with only one tooth in the male and the retromargin without tooth in the female and by the bottle-green body in in ethanol (vs. living spiders are pale green, but specimens are pale yellow, white or brownish in ethanol in almost all other genera). *Ramosatidia* gen. nov. can be distinguished from *Pristidia* by the relatively small eyes, with the PME > their diameter apart (Fig. 20E, G) (vs. PME barely > their diameter apart), from *Nusatidia* by the sternum lacking a rectangular extension beyond coxae I (cf. Fig. 20H and 10B), and from *Matidia* by the higher ocular region/carapace width ratio (approximately 2/3 vs. 1/2 in *Matidia*) (cf. Figs 20E, G and 4E, G), and femur I shorter than femur II (vs femur I longest). *Ramosatidia* gen. nov. species also can be recognised by the following characters of the copulatory organs: male palpal tibia with four apophyses (vs. maximum of three apophyses) (Fig. 19A–E); epigynal plate lacking atrium, depression, and septum (vs. with atrium or depression in *Matidia*, with septum in *Malamatidia*), fertilisation ducts relatively large, ~ 1/2 of spermathecae length (Fig. 20A–D) (vs. shorter than 1/2 of spermathecae length). All the provided characters of *Pristidia*, *Nusatidia*, and *Matidia* are according to Deeleman-Reinhold (2001) and recent clubionid papers, such as Yu et al. (2012, 2017).

##### Description.

Same as for the type species.

##### Composition.

Type species only.

##### Distribution.

China (Yunnan).

#### Ramosatidia situ

Taxon classificationAnimaliaAraneaeClubionidae

Yu & Li sp. nov.

28A09D64-DC58-58B6-90FC-2B32EEACCAD1

http://zoobank.org/EFA24D5C-4EC3-4D33-8D18-2A821F21BA34

Figs 19 , 20

##### Type material.

***Holotype***: ♂ (IZCAS-Ar34734), China: **Yunnan**: Xishuangbanna: Mengla County: Xishuangbanna Nature Reserve: Xiaolongha biodiversity preservation corridor (21°24.159'N, 101°37.178'E, 630 m), 27 June 2012, Q. Zhao leg. ***Paratype***: 1♀ (IZCAS-Ar34735), same data as holotype.

##### Other material examined.

1♀ (YHCLU0134), same data as holotype.

##### Etymology.

The specific name is derived from the Chinese pinyin *sìtū*, which means four apophyses, referring to four tibial apophyses; noun in apposition.

##### Diagnosis.

Same as for genus.

##### Description.

**Male** (holotype) (Fig. 20E–F). Total length 3.80; carapace 1.74 long, 1.24 wide; opisthosoma 2.06 long, 0.77 wide. Carapace pyriform, ocular area distinctly narrowed, in profile almost flat. Carapace yellowish white anteriorly and centrally, dark green posteriorly and marginally, without distinct pattern; fovea greenish. Eyes: in dorsal view, AER slightly recurved, PER almost straight, PER wider than AER. AME dark, other eyes light; with black rings. Eye sizes and interdistances: AME 0.09, ALE 0.08, PME 0.09, PLE 0.07, AME–AME 0.02, AME–ALE 0.04, PME–PME 0.19, PME–PLE 0.04, MOQL 0.23, MOQA 0.20, MOQP 0.38. Chelicerae yellowish green, with one tooth on promargin and five on retromargin. Labium and endites yellowish green. Sternum yellowish. Legs uniformly yellowish green. Leg measurements: Legs I and II missing, III 4.46 (1.25, 1.58, 1.24, 0.40), IV 8.47 (2.66, 2.79, 2.44, 0.58). Abdomen (Fig. 20E, F) lanceolate, surface wrinkled; dorsally uniformly yellowish green; laterally with longitudinal dark green lines; venter without distinct pattern, yellowish green centrally, bottle-green marginally; spinnerets pale green.

Palp (Fig. 19A–E): Femur and patella unmodified. Tibia relatively long, ~ 2/3 cymbium length, with four apophyses, PTA digitiform, with blunt tip, ~ 1/2 tibia length; DTA small, coniform in prolateral view, thumb-like in retrolateral view, ~ 1/3 tibia length; LTA heavily sclerotised, semi-circular with pit, like an ear in retrolateral view, ~ 1/3 tibia length; RTA ~ as long as tibia, wide proximally, narrowed distally, rough tip. Cymbium ~ 1.9× longer than wide, baso-prolaterally with a trapezoidal projection. Tegulum elongated, relatively flat, ~ 2× longer than wide, sperm duct distinct, U-shaped. Embolus, small spicule with forked tip, shorter than 1/10 tegulum length, originating apico-retrolaterally on tegulum, tip extended to apex of cymbium. Conductor ~ 1/4 tegulum length, sheet-shaped, translucent, originating from apico-retrolateral area of tegulum, covering embolic base.

**Female** (paratype IZCAS-Ar34735). Total length 3.95; carapace 1.73 long, 1.29 wide; opisthosoma 2.22 long, 0.63 wide. Similar to males but longer and darker (Fig. 20G, H). Chelicera with four promarginal teeth, retromarginal teeth lacking. Eye sizes and interdistances: AME 0.08, ALE 0.08, PME 0.07, PLE 0.07, AME–AME 0.03, AME–ALE 0.03, PME–PME 0.18, PME–PLE 0.03, MOQL 0.22, MOQA 0.19, MOQP 0.35. Legs green. Leg measurements: I 4.82 (1.44, 1.97, 0.89, 0.51), II — (1.93, 2.78, 1.35, —), III 4.06 (1.27, 1.44, 0.99, 0.36), IV missing.

Epigyne (Fig. 20A–D): Plate disc-shaped, slightly longer than wide, posterior margin not rebordered; atrium absent; spermathecae and copulatory ducts barely visible through integument. Copulatory openings small, separated by ~ 2 diameters, situated near epigastric furrow. Copulatory ducts thick, separated by ~ 1.5 diameters, ascending obliquely to spermathecae. Hyaline spermathecae large, oval, ~ 1.5× longer than wide, located anteriorly, separated by 0.5 widths. Bursae distinctly smaller than spermathecae, close together, ~ 1.29× wider than long, bursal surface hyaline, wrinkled, ribbed, pigmented and sclerotised inside. Acicular fertilisation duct relatively long, ~ 1/2 of spermathecae length, located dorso-basally on spermathecae.

##### Distribution.

Known only from the type locality.

#### Sinostidia

Taxon classificationAnimaliaAraneaeClubionidae

Yu & Li gen. nov.

1EB51659-1209-52B6-8CB0-AFFCAE80BADE

http://zoobank.org/F0401D18-0B41-487F-96DE-68EF0CB9CFE7

##### Type species.

*Sinostidiashuangjiao* Yu & Li, sp. nov.

##### Etymology.

The generic name is derived from the species’ similarity to *Pristidia* and the Latin adjective *Sino*- for Chinese, referring to the distribution of the genus. The gender is feminine.

##### Diagnosis.

**Figure 21.**
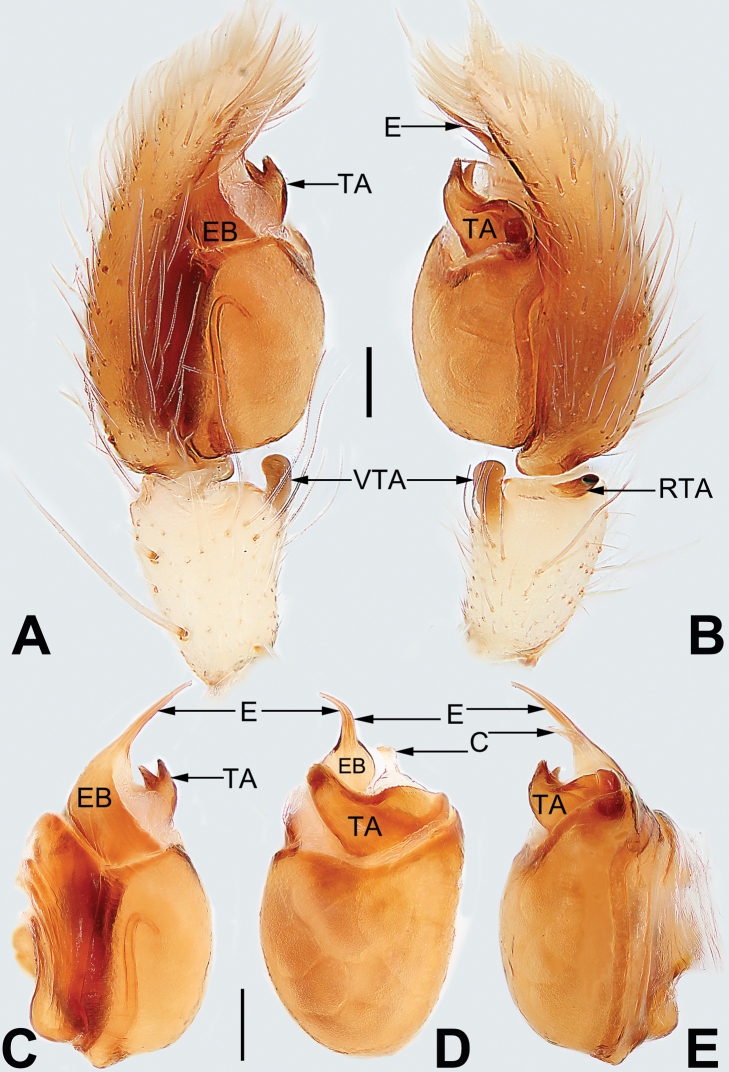
Male palp of the holotype of *Sinostidiashuangjiao* sp. nov. **A** prolateral view **B** retrolateral view **C** bulb, prolateral view **D** bulb, ventral view **E** bulb, retrolateral view. Abbreviations: C = conductor; E = embolus; EB = embolic base; RTA = retrolateral tibial apophysis; TA = tegular apophysis; VTA = ventral tibial apophysis. Scale bars: 0.10 mm (equal for **A, B**, equal for **C–E**).

**Figure 22.**
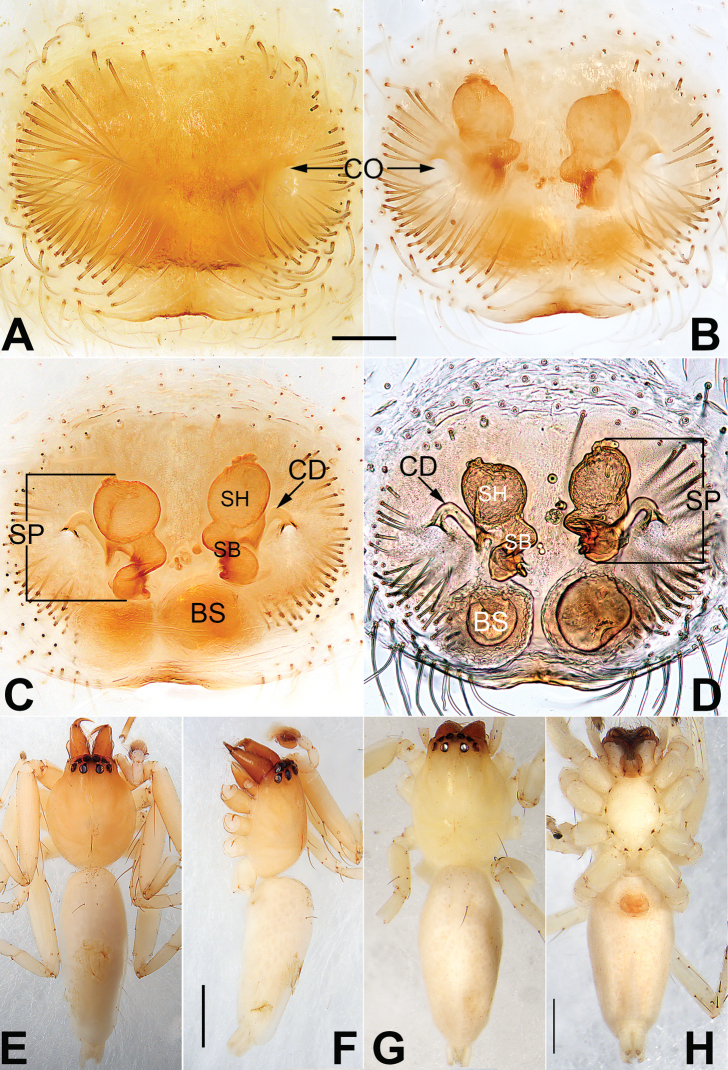
*Sinostidiashuangjiao* sp. nov., female paratype and male holotype, epigyne (**A–D**), male habitus (**E, F**) and female habitus (**G, H**) **A** intact, ventral view **B** cleared, ventral view **C** cleared, dorsal view **D** cleared, dorsal view **E** dorsal view **F** lateral view **G** dorsal view **H** ventral view. Abbreviations: BS = bursa; CD = copulatory duct; CO = copulatory opening; SB = spermathecal base; SH = spermathecal head; SP = spermatheca. Scale bars: 0.10 mm (equal for **A–D**); 1 mm (equal for **E, F**, equal for **G, H**).

**Figure 23.**
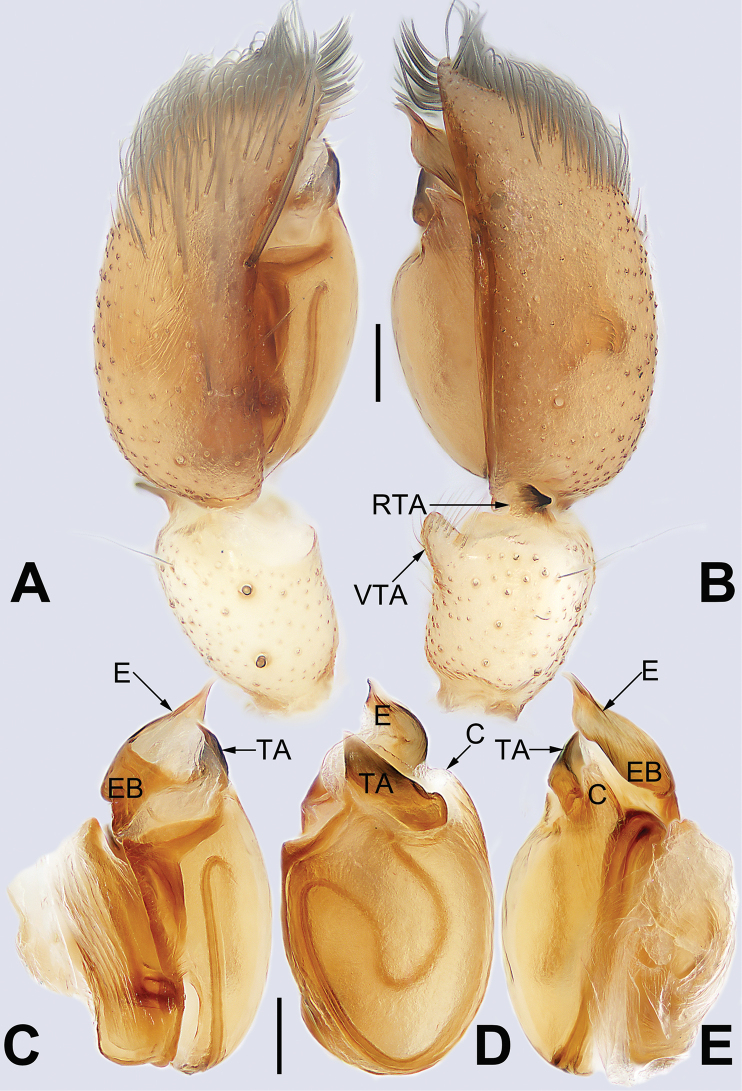
Male palp of the holotype of *Sinostidiadujiao* sp. nov. **A** prolateral view **B** retrolateral view **C** bulb, prolateral view **D** bulb, ventral view **E** bulb, retrolateral view. Abbreviations: C = conductor; E = embolus; EB = embolic base; RTA = retrolateral tibial apophysis; TA = tegular apophysis; VTA = ventral tibial apophysis. Scale bars: 0.10 mm (equal for **A, B**, equal for **C–E**).

**Figure 24.**
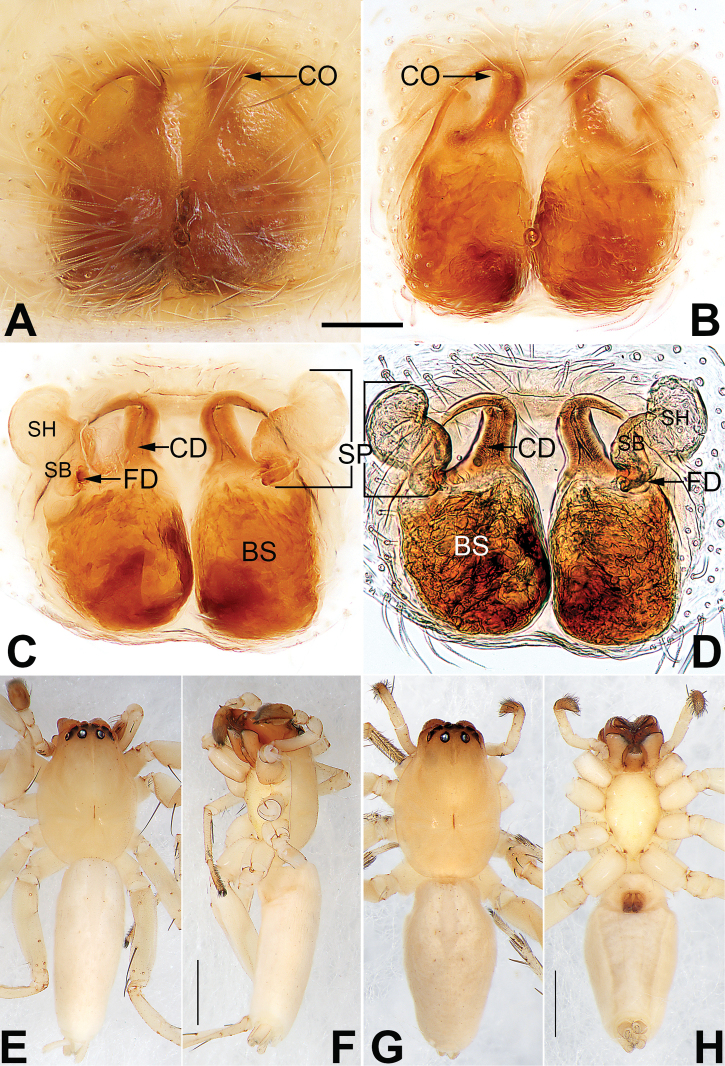
*Sinostidiadujiao* sp. nov., female paratype and male holotype, epigyne (**A–D**), male habitus (**E, F**) and female habitus (**G, H**) **A** intact, ventral view **B** cleared, ventral view **C** cleared, dorsal view **D** cleared, dorsal view **E** dorsal view **F** lateral view **G** dorsal view **H** ventral view. Abbreviations: BS = bursa; CD = copulatory duct; CO = copulatory opening; SB = spermathecal base; SH = spermathecal head; SP = spermatheca. Scale bars: 0.10 mm (equal for **A–D**); 1 mm (equal for **E, F**, equal for **G, H**).

This genus can be easily confused with *Pristidia* due to a similar appearance. *Sinostidia* gen. nov. and *Pristidia* share a similar cephalic region/carapace width ratio, relatively large eyes (PME barely > their diameter apart), tibial spination, and pale brownish body, but they can be separated by *Sinostidia* gen. nov. having promarginal teeth closer to the fang base than the retromarginal ones. The copulatory organs of *Sinostidia* gen. nov. resemble those of *Pristidia* in having a similar bulb with a sharply pointed embolus arising dorsally, hidden by the tegulum and by having a similar epigynal plate, but differ by: (1) the palpal tibia with 2 apophyses (Figs 21B, 23B) (vs. 1 apophysis); (2) the distinct and heavily sclerotised tegular apophysis (Figs 21A–E, 23C–E) (vs. tegular apophysis absent in almost all *Pristidia* species, or present but semi-transparent in *P.cervicornuta*); (3) the epigyne with a large depression or atrium (Figs 22A, B, 24A, B) (vs. depression and atrium lacking); (4) the spermathecae consist of a subglobular head and torsional base (Figs 22C, D, 24C, D) (vs. spermathecae undivided).

##### Description.

Medium-sized, with body length of males 4.5–5.0, females 4.79–5.6. Body yellow-white, legs uniformly coloured as carapace. Carapace: elongate-oval in dorsal view, pars cephalica slightly elevated above thorax, pars thoracica distinctly wider than pars cephalica; integument smooth, with sparse, erect, thin, dark bristles on pars cephalica (bristles detach easily in ethanol); yellow or pale orange, slightly darker in ocular region, without distinct pattern; fovea longitudinal, reddish. Clypeus height distinctly less than diameter of AME. Chelicerae robust, brownish red, fang furrow with three promarginal and two retromarginal teeth. Sternum yellowish, anteriorly straight, anterior and lateral margins with brown extensions fitted into intercoxal concavities; posterior region strongly protruded between coxae IV. Eyes: arranged in a compact group; AER slightly recurved in dorsal view, procurved in anterior view, AME very slightly smaller than ALE, or equal in diameter, AME closer to ALE than to each other; PER recurved in dorsal view, PME distinctly larger than PLE, PME separated by one diameter. Legs: formula usually IV, I, II, III; all tarsi scopulate; anterior metatarsi with a pair of basal ventral spines; tibiae I and II with two pairs of strong ventral spines; tibiae and metatarsi of posterior legs with more spines than anterior legs, but spination varies among different individuals. Abdomen: lanceolate, tapered posteriorly, uniformly white, dorsum with numerous indistinct patches, or anteriorly with a longitudinal, grey heart mark; venter, sides white, without distinct markings. Spinnerets: anterior lateral spinnerets short and coniform; posterior lateral spinnerets cylindrical, relatively long; anterior median spinnerets small, sandwiched between anterior lateral spinnerets and posterior lateral spinnerets.

Male palp: Femur and patella unmodified. Tibia short, no longer than 1/2 of cymbium length, with two apophyses: ventral apophysis stout, with blunt tip, typically thumb-like; retrolateral apophysis weak, shape variable, tip relatively pointed. Cymbium unmodified, ~ 1.8× longer than wide, with dense dorsal setae. Bulb elongate-oval, embolic area located distally on tegulum. Tegular apophysis large, longer than 1/2 of tegulum width, arising at ~ 1 o’clock position, gradually tapered toward apex, pointed prolatero-distally, covering embolic base. Embolus with bulky base and sharp tip, curved behind tegular apophysis, tip extended to apex of cymbium. Conductor small, situated retrolaterally on tegulum.

Epigyne: Plate with shallow, very large depression or atrium, covering > 80% of plate. Spermathecae situated anteriorly, with subglobular head and twisted base. Bursae situated posteriorly, surface wrinkled, ribbed, pigmented, sclerotised inside. Fertilisation ducts small, acicular, on distal end of spermathecal base.

##### Comments.

The large PME and the presence of a claw-shaped embolus located behind the tegulum indicate that the new genus is likely closely related to *Pristidia*. Somatic characters are either poorly delineated or variable, making the differentiation of *Sinostidia* gen. nov. and *Pristidia* difficult. However, the two new species share a distinct set of genitalic characters and can be easily separated from *Pristidia*, thus, we established a new genus to accommodate them.

##### Composition.

Two species, *Sinostidiashuangjiao* Yu & Li, sp. nov. (type species) and *Sinostidiadujiao* Yu & Li, sp. nov.

##### Distribution.

China (Yunnan).

#### Sinostidia shuangjiao

Taxon classificationAnimaliaAraneaeClubionidae

Yu & Li sp. nov.

50679D87-4597-5628-A490-1100633B595B

http://zoobank.org/972703D3-3FD3-41A7-9580-94AE85F8C561

Figs 21 , 22

##### Type material.

***Holotype***: ♂ (IZCAS-Ar34738), China: **Yunnan**: Xishuangbanna: Mengla County: Menglun Town: Menglun Nature Reserve: *Anogeissusacuminata* plantation (~ 20 years old) (21°53.993'N, 101°16.810'E, 610 m), 19 August 2007, G. Zheng leg. ***Paratype***: 1♀ (IZCAS-Ar34739, YHCLU0155), G213 roadside, Leprosy village (21°53.590'N, 101°17.296'E, 540 m), 4 May 2019, H. Yu leg.

##### Other material examined.

1♂ (YHCLU0151), same data as the paratype.

##### Etymology.

The specific name is derived from the Chinese pinyin *shuâng ji*ǎ*o*, meaning two-horned, referring to the forked tegular apophysis; noun in apposition.

##### Diagnosis.

*Sinostidiashuangjiao* sp. nov. closely resembles *S.dujiao* sp. nov. (Figs 23, 24) but can be distinguished by the shape of the copulatory organs. Males of the new species differ from those of *S.dujiao* sp. nov. by: (1) the bifid tip of the tegular apophysis represented by two tooth-shaped apophyses (vs. not bifid) (cf. Figs 21A–E and 23C–E); (2) the filiform or claw-shaped embolic tip (vs. large and conical, cf. Figs 21B–E and 23B–E); (3) the conductor is transparent and membranous (vs. thick and partly membranous) (cf. Figs 21D, E and 23D, E). The female of the new species can be differentiated from *S.dujiao* sp. nov. by lacking an atrium (vs. atrium present) (cf. Figs 22A, B and 24A, B), the copulatory openings located laterally on the epigynal plate (vs. located at anterior atrial border) (cf. Figs 22A, B and 24A, B), and by the globular bursae which are smaller than the spermathecae (vs. reniform burse distinctly larger than the spermathecae) (cf. Figs 22C, D and 24C, D).

##### Description.

**Male** (holotype) (Fig. 22E, F). Total length 4.73; carapace 1.75 long, 1.49 wide; opisthosoma 2.98 long, 1.16 wide. Carapace pyriform, ocular region distinctly narrowed; light orange, slightly darker frontally, without distinct pattern; fovea reddish. Eyes: AER slightly recurved, PER slightly wider than AER and procurved in dorsal view. AME dark, other eyes light; with black rings. Eye sizes and interdistances: AME 0.09, ALE 0.09, PME 0.12, PLE 0.11, AME–AME 0.03, AME–ALE 0.02, PME–PME 0.14, PME–PLE 0.08, MOQL 0.32, MOQA 0.28, MOQP 0.41. Chelicerae red-brown. Labium and endites light orange. Sternum yellowish white. Legs yellowish orange. Leg measurements: I 7.05 (1.95, 2.91, 1.43, 0.77), II 6.61 (1.86, 2.1, 1.44, 0.70), III 5.27 (1.55, 1.72, 1.44, 0.56), IV 7.67 (2.12, 2.59, 2.23, 0.73). Abdomen (Fig. 22E, F) lanceolate, dorsum with numerous inconspicuous patches; venter white; all spinnerets without distinct markings.

Palp (Fig. 21A–E): Retrolateral tibial apophysis claw-shaped, retrolaterally pointed, ~ 1/2 of tibia length, with curved, sharp apex; ventral tibial apophysis pointed anteriorly, thumb-like, ~ 1/3 of tibia length. Bulb oval, ~ 2× longer than wide, sperm duct indistinct in ventral view. Tegular apophysis large, as long as tegulum width, originating retrolaterally on tegulum, with bifurcated distally, covering embolic base. Embolus claw-shaped, tip extended to apex of cymbium, directed antero-prolaterally; embolic base as long as free (filamentous) part of embolus, represented by enlarged tubercle located prolaterally (~ 10 o’clock relative to tegulum). Conductor membranous, ~ 1/2 embolus length, originating distally on tegulum, base wide, apex narrowed, folded.

**Female** (paratype IZCAS-Ar34739). Total length 5.60; carapace 2.36 long, 1.74 wide; opisthosoma 3.21 long, 1.54 wide. General characters as in males, but slightly larger and lighter (Fig. 22G, H). Eye sizes and interdistances: AME 0.11, ALE 0.13, PME 0.13, PLE 0.09, AME–AME 0.06, AME–ALE 0.05, PME–PME 0.21, PME–PLE 0.11, MOQL 0.34, MOQA 0.36, MOQP 0.52. Legs uniformly white. Leg measurements: I 6.97 (1.97, 2.85, 1.38, 0.77), II 6.16 (1.70, 2.43, 1.34, 0.69), III 5.41 (1.66, 1.71, 1.50, 0.53), IV 7.97 (2.01, 2.80, 2.22, 0.95).

Epigyne (Fig. 22A–D): Plate distinctly wider than long, with shallow depression located anteriorly. Depression broad, width almost equal to the plate width, ellipsoid, ~ 1.6× wider than long; bounded by numerous, relatively long setae, sparse anteriorly, dense posteriorly and laterally. Copulatory openings small but distinct, located laterally in depression, separated by ~ 15 diameters, leading to short copulatory ducts which descend obliquely to spermathecae. Spermathecae consisting of twisted base and globular head; spermathecal heads separated by 2/3 of their diameter. Bursae globular, close together, translucent. Fertilisation ducts indistinct.

##### Distribution.

Known only from the type locality.

#### Sinostidia dujiao

Taxon classificationAnimaliaAraneaeClubionidae

Yu & Li sp. nov.

898616E8-32F3-5923-8A39-22919366A87C

http://zoobank.org/EAF566C9-A289-46EC-BB72-B6F05C8CE6B5

Figs 23 , 24

##### Type material.

***Holotype***: ♂ (IZCAS-Ar34736), China: **Yunnan**: Xishuangbanna: Mengla County: Menglun Town: Menglun Nature Reserve: 48 km landmark, seasonal rainforest (21°58.704'N, 101°19.748'E, 1080 m), 12 August 2011, G. Zheng leg. ***Paratype***: 1♀ (IZCAS-Ar34737, YHCLU0090), same data as holotype.

##### Other material examined.

1♂ (YHCLU0132), same data as the holotype.

##### Etymology.

The specific name is derived from the Chinese pinyin *dú ji*ǎ*o*, which means one-horned, referring to the unbranched tegular apophysis; noun in apposition.

##### Diagnosis.

Males of the new species are similar to those of *S.shuangjiao* sp. nov. but can be distinguished by the unbranched tegular apophysis, wide and conical embolar tip, and by the thick and partly membranous conductor (Fig. 23A–E) (vs. tegular apophysis bifurcate, embolar tip filamentous, conductor thin and entirely membranous as in Fig. 21A–E). Females of *S.dujiao* sp. nov. differ from those of *S.shuangjiao* sp. nov. by the reniform bursae larger than spermathecae (Fig. 24C, D) (vs. globular bursae smaller than the spermathecae as in Fig. 22C, D), the CO situated anteriorly and the CD located on central portion of epigyne (vs. both situated laterally in *S.shuangjiao* sp. nov. (cf. Figs 24A–D and 22A–D)).

##### Description.

**Male** (holotype) (Fig. 24E, F). Total length 4.90; carapace 2.17 long, 1.50 wide; opisthosoma 2.73 long, 0.97 wide. Carapace oval, pars cephalica distinctly narrowed; yellowish white, slightly darker frontally, without distinct pattern; fovea reddish. Eyes: in dorsal view, PER slightly wider than AER, both AER and PER slightly recurved. Eye sizes and interdistances: AME 0.12, ALE 0.12, PME 0.12, PLE 0.10, AME–AME 0.09, AME–ALE 0.03, PME–PME 0.16, PME–PLE 0.13, MOQL 0.28, MOQA 0.29, MOQP 0.43. Chelicerae red-brown, with 3 promarginal and 2 retromarginal teeth. Labium, endites light brown. Sternum yellowish. Legs yellowish white. Leg measurements: I — (1.85, —, —, —), II 6.79 (2.00, 2.25, 2.04, 0.51), III — (1.34, 1.35, 0.82, —), IV — (1.88, 2.20, 1.23, —). Abdomen (Fig. 24E, F) lanceolate, dorsally white with longitudinal, grey heart mark, extended 2/5 length of abdomen; venter white; spinnerets without distinct markings.

Palp (Fig. 23A–E). Retrolateral tibial apophysis partly membranous, ~ 1/3 of tibia length, with a pointed apex; ventral tibial apophysis digitiform and sclerotised, ~ 1/3 of palpal tibia length, with blunt tip. Bulb nearly spherical, ~ 1.6× longer than wide, sperm duct distinct, sinuate, reverse S-shaped in ventral view. Tegular apophysis triangular, longer than 2/3 tegulum width, located ventrally on distal margin of tegulum, with sharp apex, covering embolic base. Embolus thick, wrapped around tegulum dorsally, tip directed antero-mesally, embolic base nearly as long as free (conical) part of embolus. Conductor thick, small, < 1/2 embolus length, originating retrolaterally on tegulum, partly membranous distally.

**Female** (paratype IZCAS-Ar34737). Total length 4.79; carapace 2.28 long, 1.60 wide; opisthosoma 2.51 long, 1.29 wide. General colouration darker than in male (Fig. 24G, H). Eye sizes and interdistances: AME 0.10, ALE 0.14, PME 0.12, PLE 0.13, AME–AME 0.05, AME–ALE 0.06, PME–PME 0.16, PME–PLE 0.14, MOQL 0.35, MOQA 0.30, MOQP 0.44. Legs uniformly white. Leg measurements: I 5.04 (1.35, 2.10, 1.04, 0.59), II 5.06 (1.56, 1.98, 0.92, 0.60), III 4.36 (1.36, 1.48, 1.06, 0.47), IV 6.75 (2.03, 2.18, 1.95, 0.60).

Epigyne (Fig. 24A–D): Plate nearly as wide as long, with large, trapezoidal atrium as broad as plate. Atrium anteriorly bounded by margin, not rebordered posteriorly. Copulatory ducts, bursae distinctly visible through integument. Copulatory openings small, indistinct, on anterior atrial border. Copulatory ducts separated by ~ one diameter, descend posteriorly, then connect with bursae. Spermathecae with bean-shaped proximal part (head) and papilliform distal part (base), separated by ~ 2.7 widths. Bursae reniform, close together, distinctly larger than spermathecae, 1.2× longer than wide. Fertilisation ducts acicular, no > 1/4 of spermathecae length, located dorsobasally on spermathecae.

##### Distribution.

Known only from the type locality.

## Supplementary Material

XML Treatment for Malamatidia

XML Treatment for Malamatidia zu

XML Treatment for Matidia

XML Treatment for Matidia spatulata

XML Treatment for Matidia xieqian

XML Treatment for Nusatidia

XML Treatment for Nusatidia aeria

XML Treatment for Nusatidia camouflata

XML Treatment for Nusatidia changao

XML Treatment for Nusatidia mianju

XML Treatment for Nusatidia subjavana

XML Treatment for Porrhoclubiona

XML Treatment for Porrhoclubiona pteronetoides

XML Treatment for Pteroneta

XML Treatment for Pteroneta ultramarina

XML Treatment for Ramosatidia

XML Treatment for Ramosatidia situ

XML Treatment for Sinostidia

XML Treatment for Sinostidia shuangjiao

XML Treatment for Sinostidia dujiao
